# Supplementary material for: Cryo-EM structure of the deltaretroviral intasome in complex with the PP2A regulatory subunit B56γ
Source: Nat Commun. 2020 Oct 7;11:5043. doi: 10.1038/s41467-020-18874-y (PMC7542444; doi:10.1038/s41467-020-18874-y)
Supplement: Supplementary file 1 — Supplementary Information [file 41467_2020_18874_MOESM1_ESM.pdf]

Supplementary Information

Cryo-EM structure of the deltaretroviral intasome in complex with the PP2A regulatory subunit B56γ.

MICHAŁ S. BARSKI[1], JORDAN J. MINNELL[1], ZUZANA HODAKOVA[2], VALERIE E. PYE[2], ANDREA NANS[3], PETER CHEREPANOV[1,2] AND GOEDELE N. MAERTENS[1,*]

* Correspondence to: g.maertens@imperial.ac.uk

This PDF file includes:

- Supplementary Methods
- Supplementary Figures 1-16
- Supplementary Tables 1-6
- Supplementary References

Supplementary methods

Cloning of HTLV-1, HTLV-2, STLV-1 IN and human PPP2R5C constructs for prokaryotic expression

The pET28a(+)-SUMO-H6P vector was engineered by ligating annealed oligonucleotides JM1 and JM2 into *Bam*HI/*Eco*RI-digested pET28a(+)-SUMO plasmid (a gift of A. L. B. Ambrosio, Laboratório Nacional de Biociências, Campinas, Brazil). DNA sequence corresponding to residues 53 to 221 of HTLV-2 IN was PCR-amplified using primers GM130 and MB094. Following *Mfe*I and *Sal*I digest, the amplicon was ligated into EcoRI/SalI-digested pET28a(+)-SUMO-H6P. pET28(+)-SUMO-H6P-HTLV-1 IN(228-286) and (200-297) were generated by PCR amplification of the corresponding coding sequence using primers MB114 and MB054 and GNM378 and GM110, respectively. *Eco*RI- and *Sal*I-digested amplicons were ligated into similarly digested plasmid DNA. The STLV-1 MarB43 IN coding sequence was generated by codon optimizing the *integrase* gene (corresponding to nucleotides 4338-5231 of the STLV-1 MarB43 isolate genome, GenBank ID: AY590142.1) for eukaryotic expression. The codon optimized DNA was PCR-amplified using primers GNM747 and GNM748, digested with *Eco*RI and *Xho*I and ligated into similarly digested pET28a(+)-SUMO-H6P. pET28a(+)-SUMO-PPP2R5C(11-380) has been previously reported[1]. To express His_6-SUMO-LEDGF/ΔIBD-B56γ(11-380) the bases corresponding to amino acids 1-324 of *PSIP1* (gene encoding LEDGF) were amplified using primers GNM641 and GNM642, digested with *Bam*HI and *Eco*RI and ligated into *Bam*HI/*Eco*RI-digested pET28(+)-SUMO-PPP2R5C(11-380). All primers and their sequences are listed in Supplementary Table 6; all DNA constructs reported in this work were verified by sequencing.

STLV-1 IN and LEDGF/ΔIBD-B56γ site-directed mutagenesis

Two-step splicing PCR was used to generate site-directed mutants of STLV-1 IN full-length, and LEDGF/ΔIBD-B56γ(11-380). The following primer pairs were used for cloning SLTV-1 IN mutants L213A (primer GNM771, GNM770 and GNM769, GNM772), P214A (GNM773, GNM770 and GNM774, GNM769), P215A/P217A (GNM775, GNM770 and GNM776, GNM769), I216A (GNM777, GNM770 and GNM778, GNM769), E218A (GNM779, GNM770 and GNM780, GNM769), H209A (GNM769, GNM824 and GNM770, GNM829), H209/P211A (GNM769, GNM826 and GNM770, GNM830). The PCR products were then spliced together using primers GNM770 and GNM769, digested with *Eco*RI/*Xho*I and ligated into similarly digested pET28(+)-SUMO-H6P. The following primer pairs were used to clone LEDGF/ΔIBD-B56γ mutants E78A/T81A/H82A (GNM283, GNM641 and GM142, GNM750), R143A (GNM283, GNM751 and GM142, GNM752), N83A (GNM283, GNM844 and GM142, GNM845), R84A (GNM283, GNM833 and GM142, GNM834), P148A (GNM283, GNM837 and GM142, GNM838). The PCR products were then spliced together using primer pairs GM142 and GNM283, digested with *Eco*RI/*Sal*I and ligated into *Eco*RI/*Sal*I-digested pET28(+)SUMO-LEDGF/ΔIBD-*PPP2R5C*(11-380).

Cloning of full length *PPP2R5C* for eukaryotic expression.

The full length *PPP2R5C* gene was PCR-amplified from HeLa cDNA using primers GNM380 and GNM367, digested with *Age*I and *Sal*I and ligated into *Age*I/*Xho*I-digested pQFlag puroR[2]. Sequencing of the resulting construct established that cloned cDNA corresponds to isoform 2 of *PPP2R5C* gene transcript. Point mutations were introduced by splicing PCR as described above with the difference that the primers annealing to the 5' and 3' ends respectively were JM3 and GNM380.

Crystallisation of HTLV-2 IN/CCD (53-221)

Expression was conducted in *E. coli* Rosetta-2 (DE3) pLacI cells (Novagen) in Terrific Broth (TB, Melford). Cells were grown to the OD_{600} of 2.0 at 30°C, followed by 30-min incubation at 18°C and induction by 0.01% IPTG at 18°C overnight. The pelleted cells were disrupted by sonication in 0.5 M NaCl, 10 mM imidazole, 50 mM Tris-HCl pH 7.4, 1 mM phenylmethylsulfonyl fluoride (PMSF). The soluble protein, captured on His-Select nickel immobilized metal affinity chromatography (IMAC) resin (Sigma-Aldrich, UK), was extensively washed with buffer containing 10 mM imidazole, 0.5 M NaCl, 50 mM Tris-HCl pH 7.4. Following elution from IMAC with the wash buffer supplemented with 200 mM imidazole, cleavage was conducted at 4°C overnight with HRV 3C protease (using 1 mg protease per 20 mg protein) in presence of 5 mM DTT. Subsequently, the protein was diluted 5-fold with salt-free buffer to achieve a final NaCl concentration of 100 mM and subjected to cation-exchange chromatography on a 5-ml high-performance SP column (GE Healthcare, UK). Fractions containing IN/CCD were pooled and injected onto an S200 16/60 size-exclusion column (GE Healthcare, UK) with 50 mM Tris-HCl pH 7.4, 0.5 M NaCl, 2 mM DTT as running buffer. Positive fractions were collected and concentrated in a 10-kDa MWCO Vivaspin centrifugal ultrafiltration device (Sartorius) to a volume of 1 mL. This was then diluted two-fold in ice-cold ultra-pure water and concentrated further to yield 15 mg/mL HTLV-2 IN/CCD in 250 mM NaCl, 1 mM DTT, 25 mM Tris-HCl pH 7.4.

The sample prepared as above was used to set up 960 sitting-drop vapour diffusion sparse-matrix crystallisation conditions. Initial screens were set up using the Mosquito liquid dispenser (SPT Labtech), with 800 nL drops consisting of a 1:1 ratio of protein-to-precipitant and allowed to incubate at 18°C. Final crystal growth was achieved by hanging-drop vapour diffusion in 2 μL drops consisting of 2:1 ratio of 21 mg/mL HTLV-2 IN/CCD-to-precipitant.

For the CCD-Mg^{2+} complex, the precipitant solution comprised of 100 mM Tris-HCl pH 8.5, 14% polyethylene glycol (PEG) 8,000 and between 100 mM and 250 mM $MgCl_2$. For the CCD-Ca^{2+} complex, the precipitant used was: 100 mM Tris-HCl pH 8, 18% PEG 6K, 250 mM $CaCl_2$. Maximum crystal growth in the condition containing $MgCl_2$ and $CaCl_2$ appeared after 48 h and 3 months at 18°C incubation, respectively. Crystals grew as large elongated hexagons or cubes, with the lengths of up to 0.5 mm.

Crystals, cryoprotected in a step-wise fashion with reservoir solution supplemented with 10%, then 20% glycerol (v/v), were cryo-cooled by plunging into liquid nitrogen. Native data collection was carried out at beamline I03 for the IN/CCD-Mg^{2+} and I04 for the IN/CCD-Ca^{2+} at Diamond Light Source (Didcot, UK). A 0.976 Å wavelength beam was used to collect diffraction images with 0.01 s exposures under 0.1° oscillation angle, over a total rotation angle of 360°. Crystals diffracted to a maximum resolution of approximately 2 Å. Data were indexed and integrated in Xia2[3] using XDS[4], which identified datasets belonging to two unique space groups. Datasets were scaled and merged in CCP4i2[5] Aimless[6], with the resolution limit adjusted accordingly until satisfactory signal-to-noise and completeness were reached. Unit cell composition was estimated from Matthew's coefficient. Phases were obtained by molecular replacement in PHASER[7] through the PHENIX software suite[8]. The mouse mammary tumour virus (MMTV) IN/CCD (PDB ID: 5CZ1) was used as a search model and resulted in a solution with a TFZ score of 50. PHENIX Autobuild[9] was successful in building the majority of residues in all chains. The remaining residues were added manually in Coot and the model was refined in Refmac 5.8.

Crystallisation of HTLV-1 IN/CTD (228-286)

The HTLV-1 IN/CTD construct that led to good quality highly diffracting crystals encodes for residues 228-286 and is further referred to as the IN/CTD. Expression was conducted as described above for HTLV-2 IN/CCD. Extraction was performed in 50 mM Tris-HCl pH 7.4, 0.5 M NaCl, 10 mM imidazole, 1 mM PMSF, through sonication of the resuspended. This was then clarified by centrifugation at 50 000 *g* for 15 min at 4ºC. Once bound to the IMAC resin, the sample was washed with 100 mL of 50 mM Tris-HCl pH 7.4, 2 M NaCl, 10 mM imidazole in order to dissociate the contaminating nucleic acid. Following elution in 50 mM Tris-HCl pH 7.4, 0.5 M NaCl, 200 mM imidazole, digestion of the His_6-SUMO-His_6 tag was performed with HRV 3C protease overnight at 4ºC in presence of 5 mM DTT. Due to the apparent cold-induced precipitation of HTLV-1 IN/CTD, all the following steps were performed at room temperature. Ion-exchange chromatography was conducted with a high-performance SP column (GE Healthcare, UK) with the sample diluted to 150 mM NaCl concentration for binding. Gel filtration of positive fractions was carried out on Superdex-200 16/60 size-exclusion column (GE Healthcare, UK) with 50 mM Tris-HCl pH 7.4, 300 mM NaCl. Positive fractions were pooled, diluted 1:1 with ultrapure water to yield final buffering conditions of 25 mM Tris-HCl pH 7.4, 150 mM NaCl, and supplemented with 2 mM DTT. The sample was concentrated in a 3K MWCO Vivaspin ultrafiltration device (Sartorius) to 14 mg/mL.

A Mosquito liquid dispenser was used to set up 960 crystallisation conditions in sitting-drop plates. Successful crystallisation was observed in several conditions, of which the most promising one comprised 1.3 M ammonium tartrate dibasic, 0.1 M bis-tris propane (BTP)-HCl pH 7.0. Rhombohedral crystals appeared after three days. Optimisation led to the selection of 1.1 M ammonium tartrate dibasic as the optimal condition for crystal growth in the hanging-drop format, and the protein:precipitant ratio of 2:1. Apparent over-nucleation, affecting crystal

size, was resolved by microseeding. Seeds were prepared by maceration of medium-size crystals in mother liquor containing the same crystallisation components as the target condition. Seeds were then collected, diluted 1,000-fold in the reservoir solution and kept on ice. Streak-seeding was performed immediately after.

Crystals were harvested and cryoprotected in a step-wise fashion with reservoir solution supplemented with 10%, then 20% glycerol (v/v). Crystals were then cryo-cooled in liquid nitrogen. Native data collection was carried out at beamline I04 at Diamond Light Source (Didcot, UK). A 0.979 Å wavelength beam was used to collect diffraction images with 0.01 s exposures per 0.1^{o} oscillation angle, over a total rotation angle of 360°. Crystals diffracted to a maximum resolution of 1.14 Å. Data were indexed and integrated in XDS[4] via Xia2[3]. Datasets were scaled and merged in CCP4i2[5] using Aimless[6], with the resolution limit adjusted accordingly until satisfactory signal-to-noise and completeness were reached. Unit cell composition was estimated from Matthew's coefficient. Phases were obtained by molecular replacement using PHASER[7] through the PHENIX software suite[8]. Using the MVV IN/CTD structure as a replacement model (PDB code 5LLJ) resulted in a single solution with a TFZ score of 30.1. PHENIX Autobuild[9] was successful in building the majority of residues in all chains. The remaining residues were added manually in Coot and the model was refined in Refmac 5.8.

Crystallisation of HTLV-1 IN (200-297) : B56γ complex

The PP2A regulatory subunit B56γ (11-380) (further referred to as B56γ) was expressed and purified as described previously[10]. Expression and purification of HTLV-1 IN (200-297) was performed as described above for the 228-286 construct. HTLV-1 IN (200-297) did not display cold-induced precipitation properties and was therefore kept cold throughout the purification.

Freshly purified B56γ and HTLV1 IN (200-297) were concentrated to 5 mg/mL, and combined at a 1:1 molar ratio, followed by incubation on ice for 10 minutes. Samples were dialysed against ice-cold buffer (50 mM Tris-HCl pH 8.0, 200 mM NaCl, 2 mM DTT) overnight at 4˚C. The complex was purified by gel filtration in the above buffer and the peak fractions were confirmed to contain both proteins by SDS-PAGE. The HTLV-1 IN (200-297)-B56γ complex was concentrated to 26 mg/mL in a 10 kDa MWCO ultrafiltration device (Vivaspin). A 100 nL sample and 100 nL crystallisation buffer drops were set up in a sitting-drop vapour diffusion format using the mosquito liquid-handling robot. Initial crystallisation was observed in 0.1 M Na/KPO_4 pH 6.2, 25% v/v 1,2-propanediol, 10% v/v glycerol. Optimisation of this condition led to crystallisation in the hanging-drop vapour-diffusion format in 0.1 M Na/KPO4 pH 6.2, 20% 1,2-propanediol, 10% glycerol. In order to increase crystal size and control nucleation, microseeding was conducted with crystals from the above condition. Crystals suspended in mother liquor were macerated with the crystal crusher tool (Hampton Research), and a 1:100 dilution of the seed stock was streak-seeded onto new drops. The resulting crystals were thin plates, approximately 50 μm by 50 μm.

Data were collected on beamline I24 at Diamond Light Source (Didcot, UK). Reflections were indexed using Xia2[3] in 3dii mode[4]. In order to increase the signal-to-noise ratio, two wedges of images with the highest Bragg reflection number were extracted from the original dataset and re-indexed together in space group $P4_32_12$. Phaser[7] was used for phasing by molecular replacement using the B56γ structure (PDB ID: 2JAK) as the template. PHENIX Autobuild[9] was successful in building the majority of residues in all chains. The remaining residues were added manually in Coot and the final model was refined in Refmac 5.8.

Tissue culture and Flag-immunoprecipitation (IP)

Human Embryonic Kidney SV40 large T cells (further referred to as 293T) were cultured in Dulbecco's Modified Eagle Medium (Sigma) supplemented with 10% foetal bovine serum (Sigma), 100 IU/mL penicillin, and 100 μg/mL streptomycin (Sigma) and grown in a humidified incubator at 37°C with 5% CO_2. 293T cells were transfected with 20 μg plasmid DNA by calcium phosphate precipitation as previously described[11]. Thirty-six hours post-transfection, cells were harvested and moved to ice. All further procedures were done on ice or at 4°C. Cells were lysed in 5 volumes of PP2A IP buffer (20 mM Tris-HCl pH 8.0, 0.1% (v/v) Nonidet P-40, 150 mM NaCl, 3 mM EDTA, 3 mM EGTA) supplemented with Complete EDTA-free protease inhibitor cocktail (Roche), left on ice for 10 min and cellular debris were removed by centrifugation at 16,000 *g* for 30 min. Protein concentration in the extract was determined using the DeNovix spectrophotometer (assuming 1 A_{280} corresponds to 1 mg/ml protein) and 3 mg of total protein for each sample in a volume of 400 μl was allowed to bind to 25 μl pre-washed anti-Flag agarose beads (Sigma) by end-over-end rocking at 4°C. Beads were washed 4 times in 1 ml IP buffer. After removing all remaining liquid from the beads, proteins were eluted by boiling in 30 μl of Laemmli buffer. To detect BUBR1, CHK2, and Cα, in the protein lysate, 30 μg total protein (equivalent with 1% used for the IP) was loaded on gel. For detection of Flag-B56γ and Aα, 10 μg of lysate was loaded. Ten μl eluate was separated on an 11% SDS-PAGE denaturing gel, proteins were electrotransferred onto nitrocellulose. Membranes were blocked in 5% milk/PBS and probed with the following antibodies: horse radish peroxidase (HRP) conjugated mouse anti-Flag antibody (1: 2,000, clone M2, Sigma, A8592), rabbit anti-BUBR1 (1:1,000, Bethyl Laboratories, A300-365A-T), rabbit anti-CHK2 (1:200, Santa Cruz Biotech, H-300, sc-9064), rat anti-Aα (1:2,000 clone 6G3, Insight Biotechnology, sc-56954), mouse anti-Cα (1:2,000, clone 46, Becton-Dickinson, 610556). All antibodies were diluted in 5% milk/PBST (PBS supplemented with 0.1% Tween-

20). HRP conjugated donkey anti-rabbit (GE Life Sciences, GE NA934), rabbit anti-rat (Abcam, ab6734) and rat anti-mouse (Abcam, ab131368) were used at 1:2,000 dilution. Detection was done using ClarityMax ECL (BUBR1 and CHK2) or Clarity ECL (all remaining proteins) reagents from BioRad and imaged on Azure600. All IP experiments were done in triplicate.

Supplementary figures

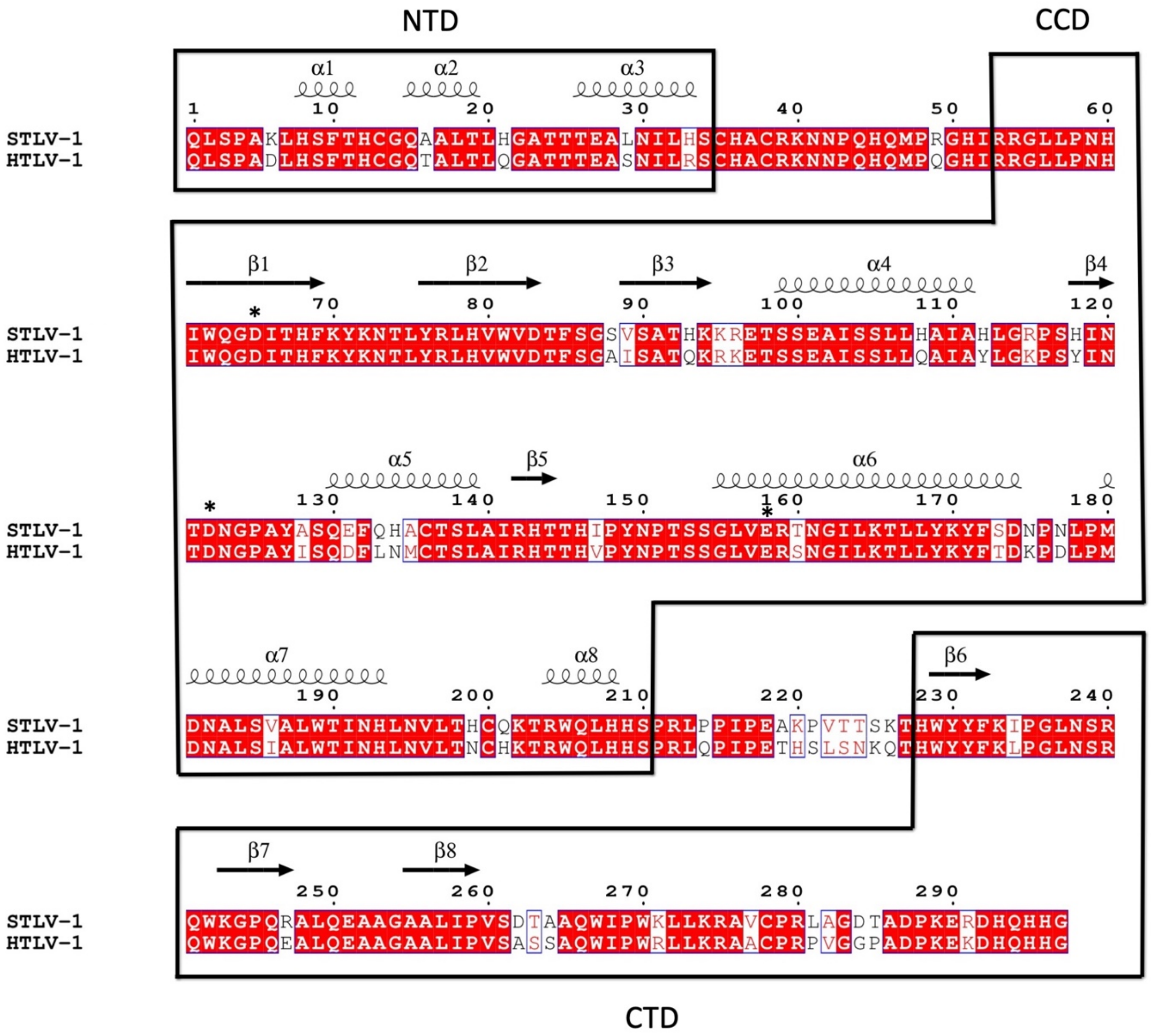

Supplementary Figure 1 | Sequence Alignment of STLV-1 MarB43 and HTLV-1 INs. N-terminal (NTD), catalytic core (CCD) and C-terminal (CTD) domains are indicated with black boxes. Residue identity is indicated with red background, similarity with red lettering. Residues forming the catalytic triad DDE are indicated with asterisks. The STLV-1 MarB43 and HTLV-1 IN sequences exhibit 83% identity and 92% similarity. Alignment was conducted with CLUSTALW[12] and visualised in ESPRIPT[13].

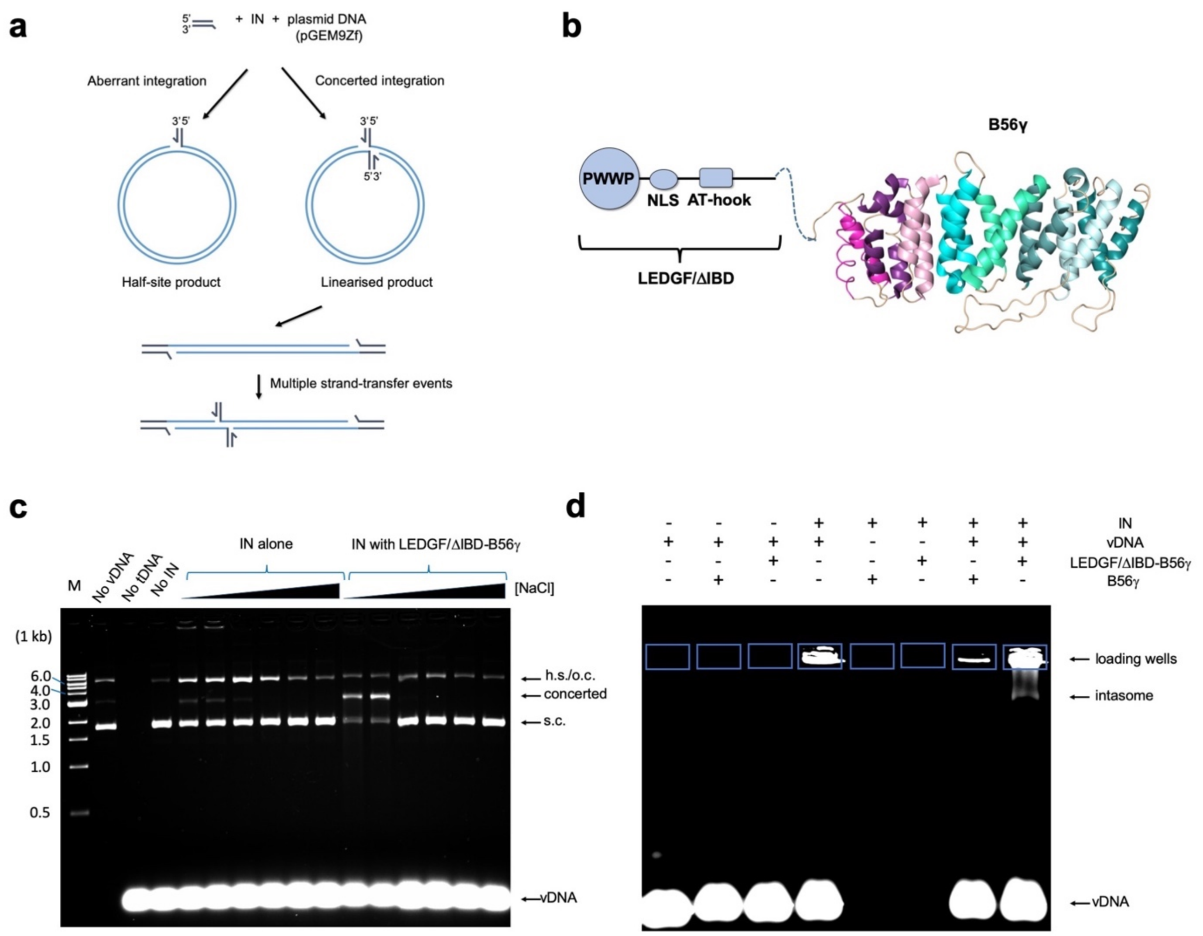

Supplementary Figure 2 | Optimisation of STLV-1 intasome activity and assembly. a, IN strand-transfer activity can be measured by means of an assay where LTR mimics (vDNA) and target DNA (tDNA, a supercoiled plasmid) are mixed with recombinant STLV-1 IN. A linearised plasmid product corresponds to integration of both vDNA ends inserted. **b**, Schematic of LEDGF/ΔIBD-B56γ structure. **c**, STLV-1 strand-transfer activity was measured at different NaCl concentrations during co-incubation with vDNA in presence or absence of LEDGF/ΔIBD-B56γ. The NaCl concentrations from left to right: 60 mM, 100 mM, 200 mM, 300 mM, 400 mM, 500 mM. M stands for the marker ladder (NEB, 1kb), h.s – half-site integration, o.c. – open circular, s.c. – supercoiled. Although residual activity of STLV-1 IN is visible without LEDGF/ΔIBD-B56γ, activity with the binding partner, as previously shown for B56γ on its own[1], is considerably enhanced. NaCl concentrations above 100 mM have a negative impact on strand-transfer activity. **d**, EMSA assay showing the dependence of successful STLV-1 intasome formation on the presence of LEDGF/ΔIBD-B56γ. B56γ alone is not sufficient to lead to stable intasome assembly. Atto680-labelled vDNA (30 bp) was used to visualise the DNA on a 3% low melting point agarose gel. Gels shown are representative of at least three independent replicates.

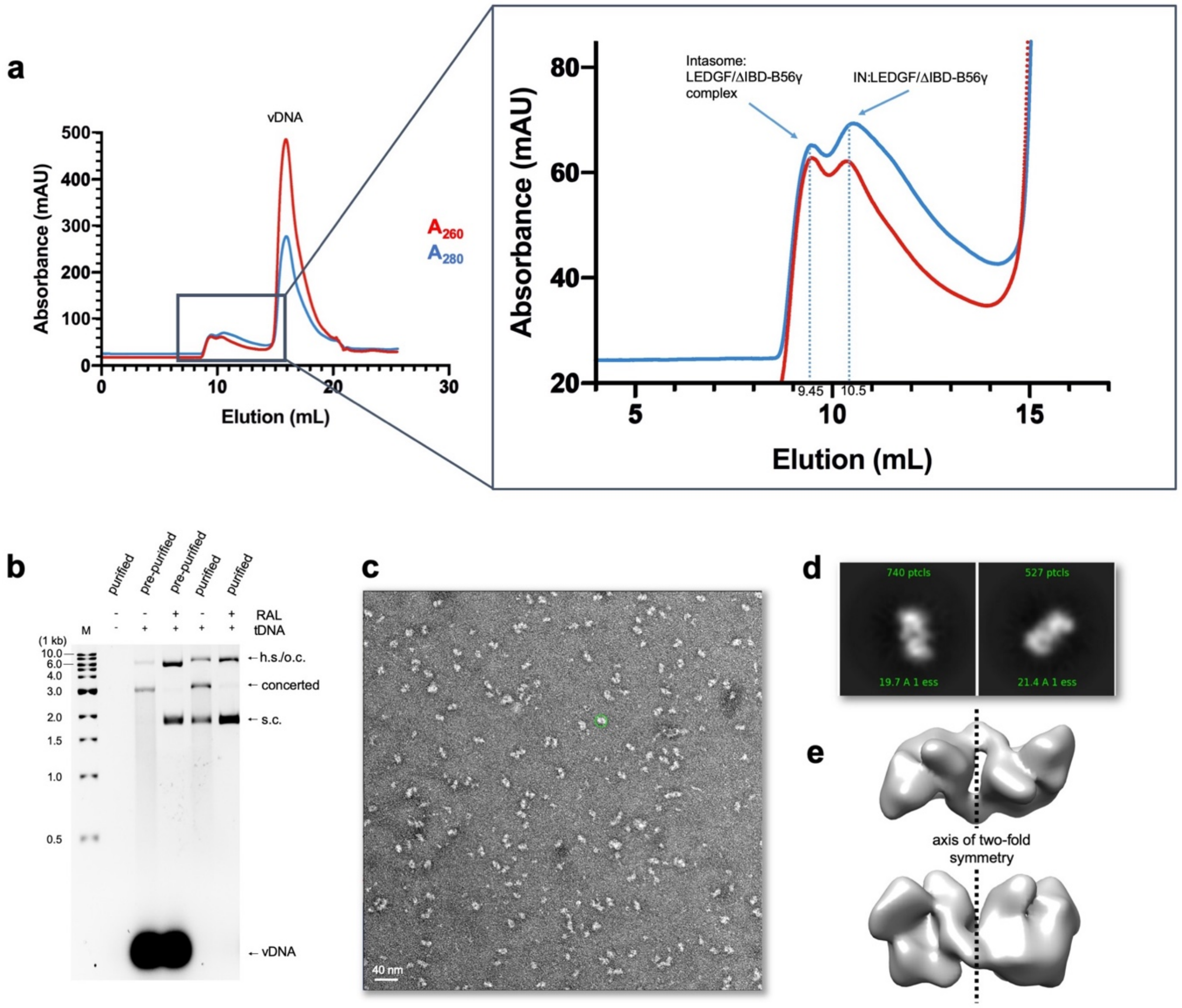

Supplementary Figure 3 | Assembly of STLV-1 intasomes for electron microscopy. a, Purification of STLV-1 intasome by size exclusion chromatography (SEC). Two high-molecular weight peaks corresponding to the assembled STLV-1 intasome eluting at 9.45 mL and the smaller IN: LEDGF/ΔIBD-B56γ complex, devoid of vDNA, eluting at 10.5 mL. The blue trace shows absorbance recorded at 280 nm, the red trace absorbance at 260 nm. **b**, Analysis of strand-transfer activity associated with crude and SEC-purified intasome fractions in presence or absence of target DNA (tDNA) and the strand-transfer inhibitor raltegravir (RAL). Products were resolved on a 1.5% agarose gel, stained with ethidium bromide. M stands for the marker ladder (NEB, 1kb); h.s., half-site integration; o.c., open circular; s.c., supercoiled. **c**, Electron micrograph of the intasome SEC fraction negatively stained with uranyl acetate. An example of a particle is circled in green, measuring ~150 Å. **d**, Examples of 2D class averages of 22,000 negatively-stained intasome images. **e**, Orthogonal views of a 3D reconstruction obtained from 8,790 negatively-stained STLV-1 intasome images selected by

reference-free 2D classification; two-fold symmetry axis is indicated. Data shown is representative 3 of independent biological replicates.

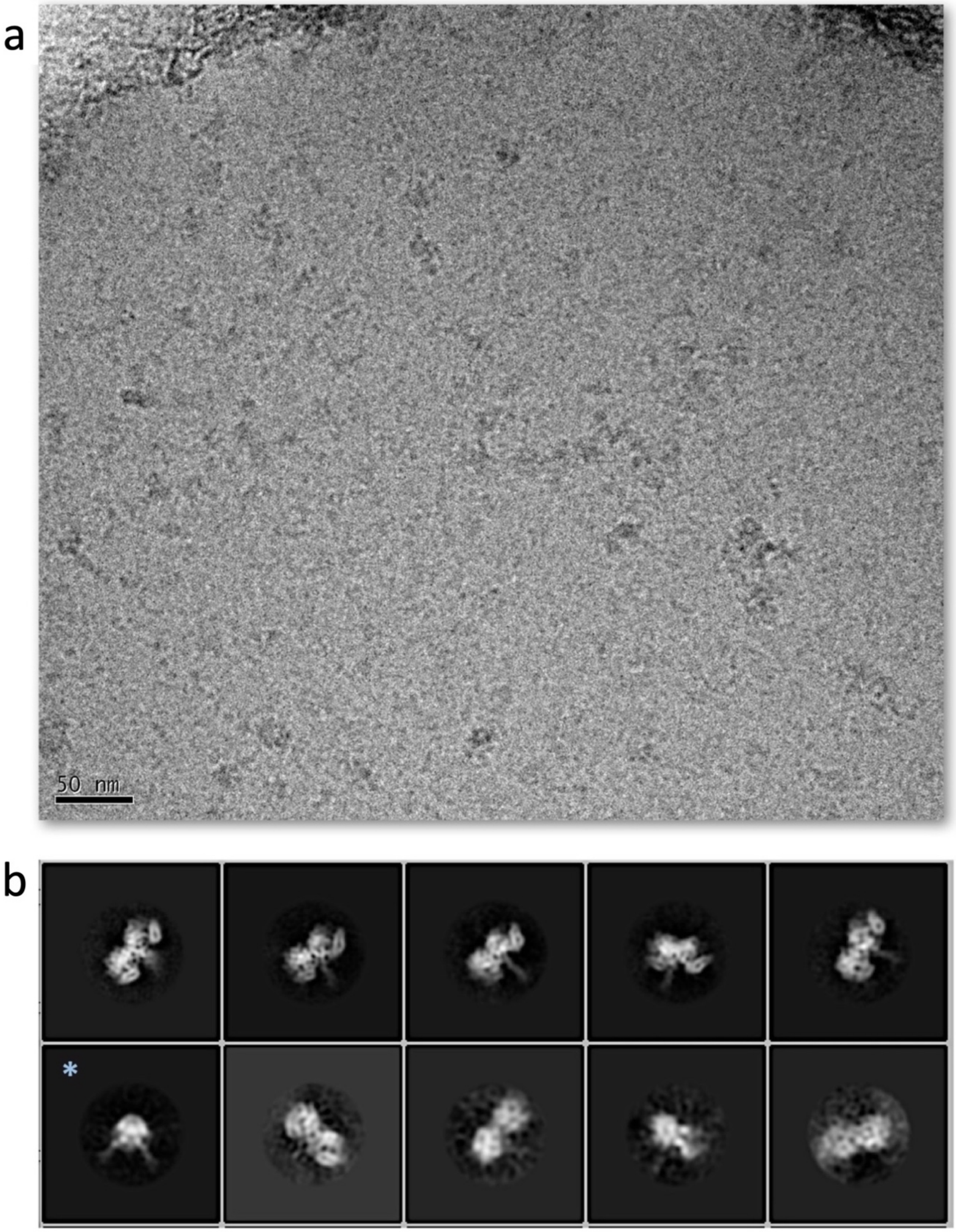

Supplementary Figure 4 | Initial Cryo-EM analysis of the STLV-1 intasome. a, An example of cryo-EM micrograph (open holes) showing STLV-1 intasome particles used for

obtaining initial 2D classes (**b**). Most classes represent the “side” view of the intasome while the class marked with an asterisk represent the “through” view.

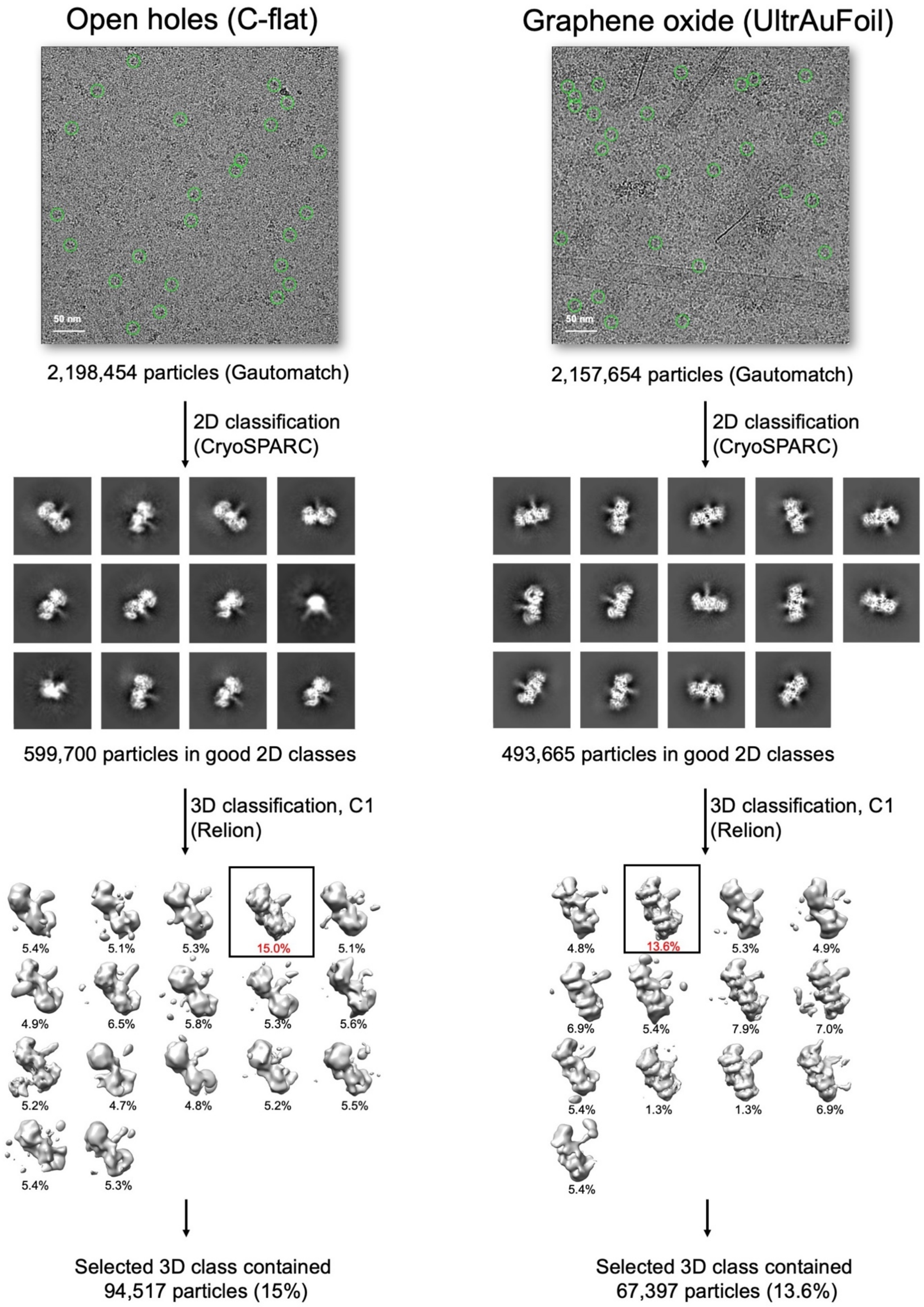

Supplementary Figure 5 | Schematic of cryo-EM image processing. Open hole (OH) and graphene oxide (GO) datasets were processed separately prior to merging to alleviate severe

anisotropy due to strong (but complementary) preferential particle orientations on OH and GO grids (Supplementary Figure 6, Supplementary Table 5). Details are given in Materials and Methods.

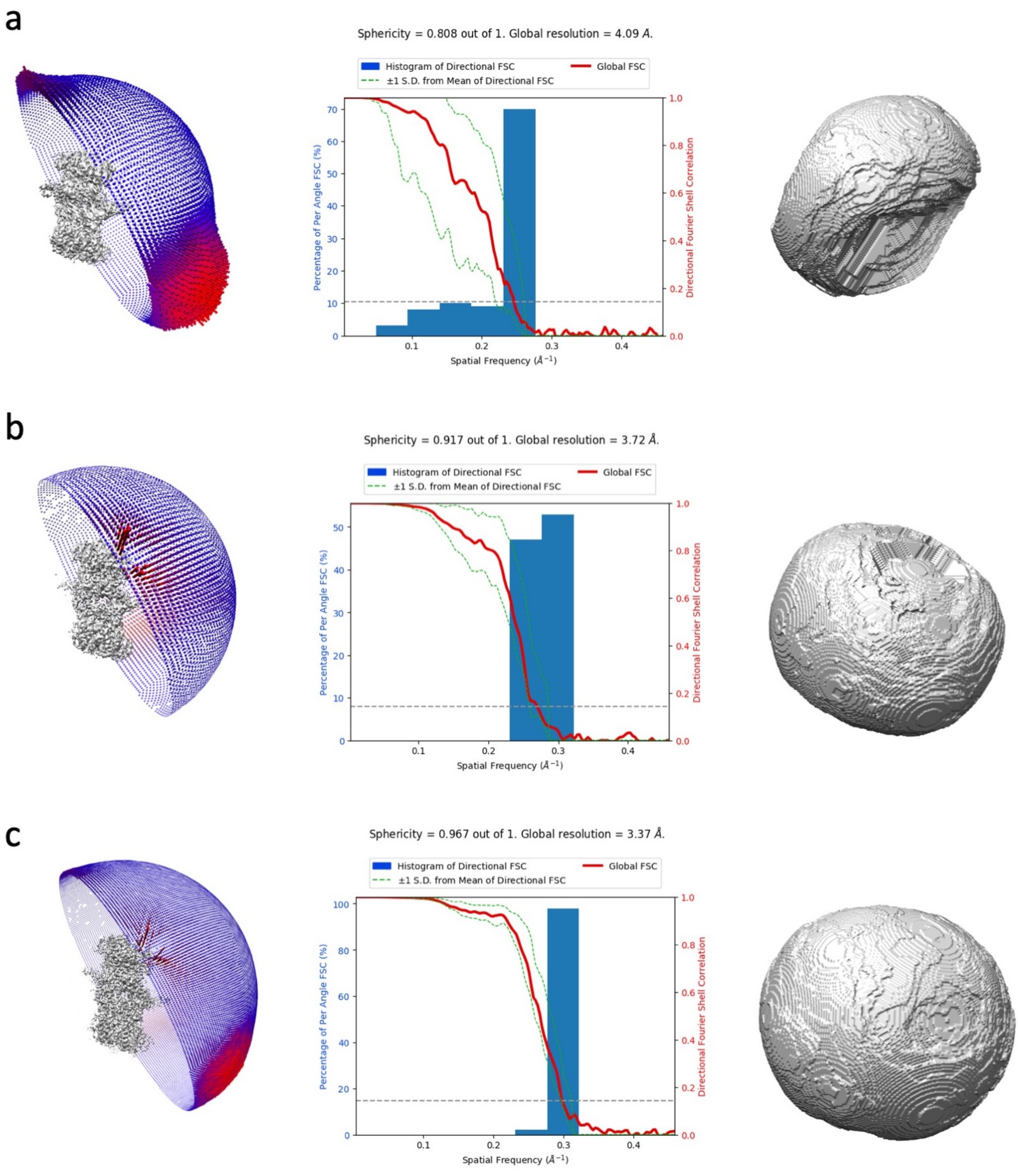

Supplementary Figure 6 | Orientational bias and anisotropy analysis. Orientational bias was analysed for each of three datasets, corresponding to collections in open holes **(a)**, graphene oxide-coated grids **(b)**, and the merged final dataset **(c)**. The observed Euler angles (left), combined 2D FSC and 3D FSC histogram (middle) and binarized 3D FSC volumes[14]

(right) are shown. Reconstructions from both OH and GO datasets suffer from considerable anisotropy, as indicated by presence of directions of low resolution (ranging between 12 and 4 Å for the map reconstructed from OH data) and non-spherical binarized 3D FSCs (**a, b**). Merging both datasets enriched the Euler angle distribution, significantly reducing the anisotropy and increasing the quantified sphericity score of the reconstruction (**c**).

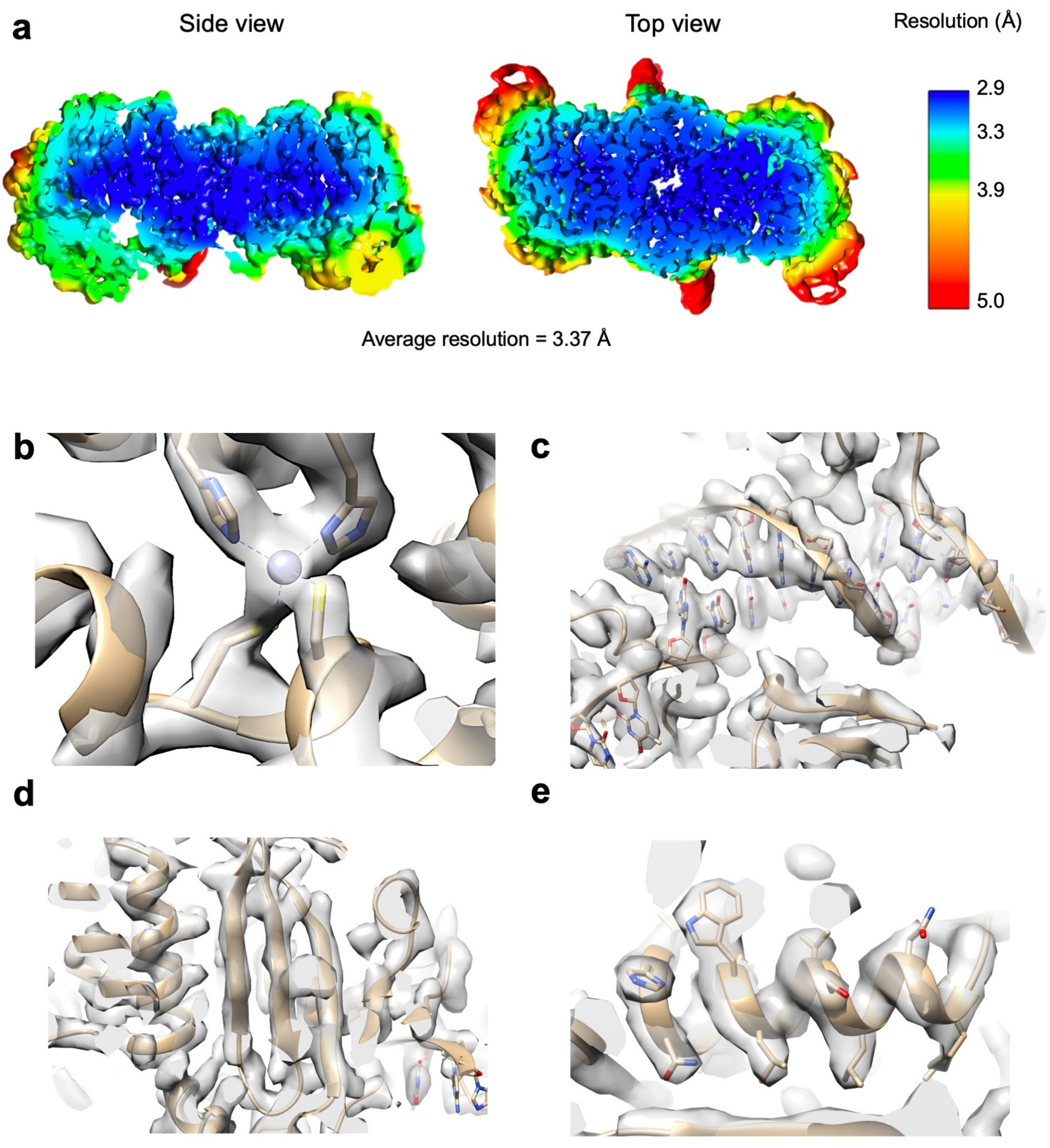

Supplementary Figure 7 | Analysis of the reconstructed STLV-1 map. a, Local resolution of the reconstruction is mapped onto the density showing an average resolution of 3.37 Å, with the highest resolution (blue) of 2.9 Å around the core of the molecule (including the active site), and the lowest (red) in the outside helices of B56γ and terminal bases of vDNA. **b**, *Ab initio* building of the STLV-1 IN/NTD domain. Density for the Zn^{2+} ion (blue sphere) is clearly

resolved, together with the HHCC-binding motif. **c**, Building of the vDNA double helix. The double strand is then resolved in the intasome active site (left). **d**, Central β-sheet of the STLV-1 IN/CCD once fitted into the density. Clear separation of β-strands density is indicative of a sub-4 Å cryo-EM reconstruction. **e**, High resolution of the intasome reconstruction allows for unambiguous placement of the majority of amino acid side chains; pictured is the IN/CCD helix α7.

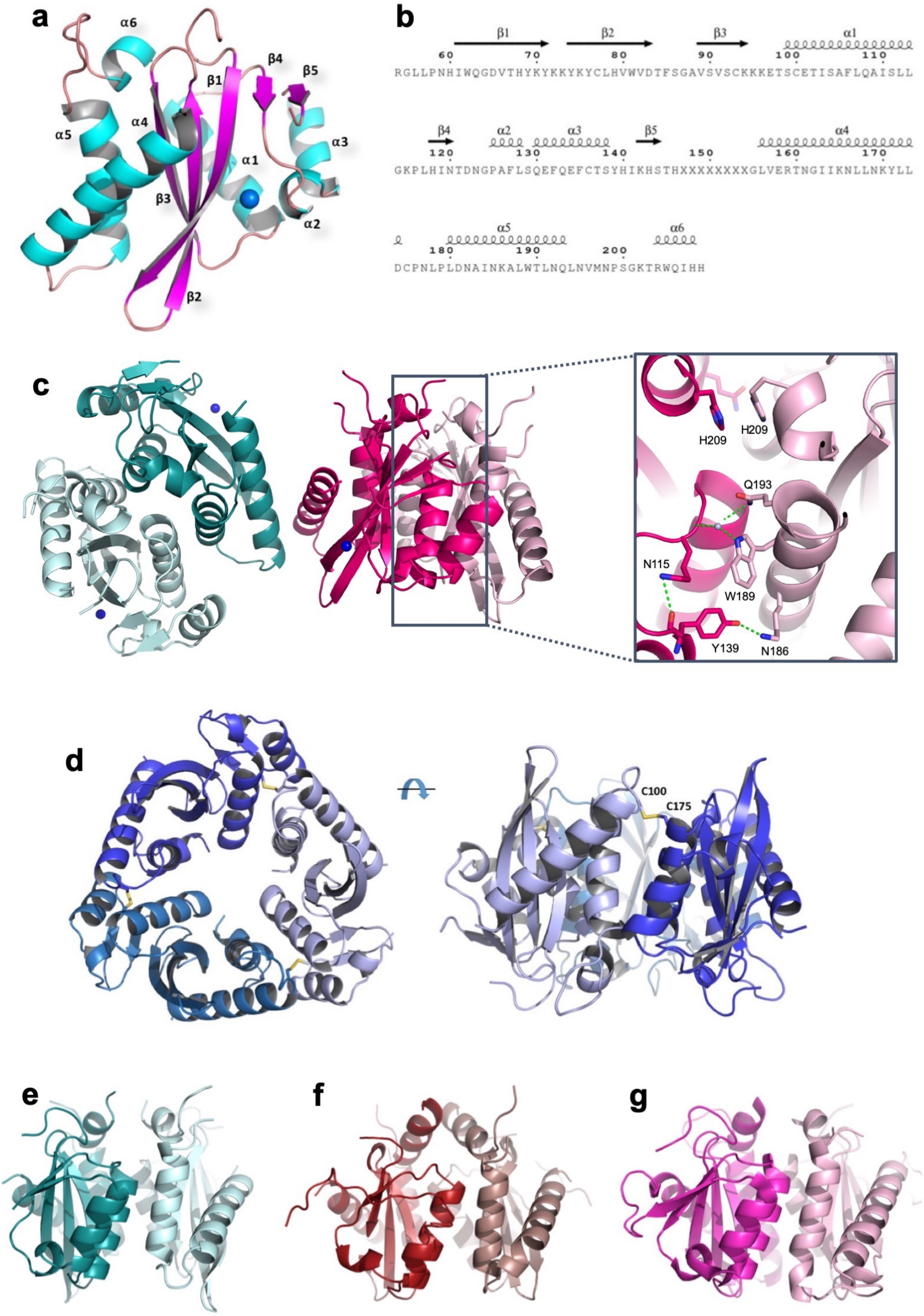
a
α6
α5
α4
β1
β4
β5
α3
α1
β3
α2
β2
b
β1
β2
β3
α1
60
70
80
90
100
110
RGLLPNHIWQGDVTHYKYKKYKYCLHVWVDTFSGAVSVSCKKKETSCETISAFLQAISLL
β4
α2
α3
β5
α4
120
130
140
150
160
170
GKPLHINTDNGPAFLSQEFQEFCTSYHIKHSTHXXXXXXXXGLVERTNGIIKNLLNKYLL
α5
α6
180
190
200
DCPNLPLDNAINKALWTLNQLNVMNPSGKTRWQIHH
c
H209
H209
Q193
N115
W189
Y139
N186
d
C100
C175
e
f
g

Supplementary Figure 8 | X-ray crystal structures of HTLV-2 IN/CCD. a, Analysis of overall HTLV-2 IN/CCD structural features. Secondary structural features are shown as α-helices in cyan and β-strands in pink. The complexed magnesium atom is shown in blue. **b**, Amino acid sequence for HTLV-2 IN/CCD with the secondary structure topology overlaid. **c**, Crystal packing in the C2 space group reveals two distinct dimerisation interfaces, including the stable, canonical, form indicated with the grey rectangle. The interaction interface of the canonical dimer is shown in more detail in the inset (right). Residues important for the stabilisation of this interface are shown as sticks. Water molecules involved in these interactions are shown as light blue spheres and putative polar interactions as green dotted lines. **d,** We also crystallised an unusual trimeric assembly of IN/CCD formed between symmetry-related chains in the $P4_332$ space group. Although the interface buries 1,627 Å^2 of surface area (compared with 874 Å^2 buried by the canonical dimer), IN/CCD exists only as a dimer in solution (Supplementary Figure 9). The disulphide bridges and the cysteines involved in trimerisation are indicated in yellow. **e-g**, Comparison of the highly structurally-conserved canonical dimer interfaces in IN/CCD structures of related retroviral genera. The panels represent CCD structures from (**e**) HTLV-2, (**f**) HIV-1 (PDB ID: 5KRT), and (**g**) RSV (PDB ID: 1C1A) IN.

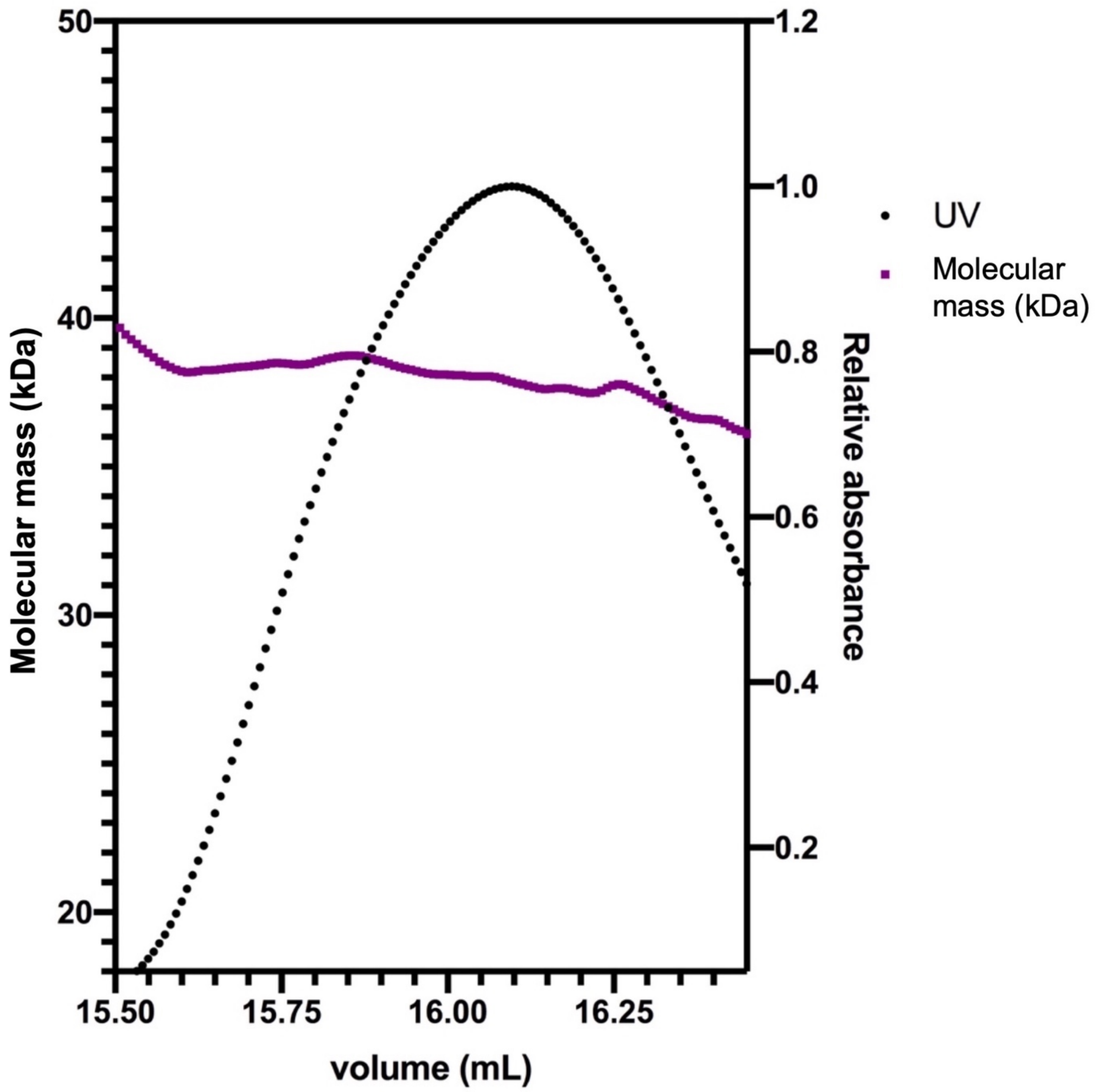

Supplementary Figure 9 | SEC-MALLS analysis of HTLV-2 IN/CCD. A purified sample of HTLV-2 IN/CCD (residues 53-221) was analysed in a SEC-MALLS experiment to more precisely ascertain the molecular mass (MW) of its oligomeric state in solution. UV absorption was recorded and showed a single elution peak at 16.1 mL. The molecular mass for the peak was calculated in the ASTRA software (Wyatt Technology, UK) from light scattering and differential refractive index. The calculated molecular mass of 38.13 kDa would correspond to a dimer (mass of a monomer being 19.2 kDa) of IN/CCD.

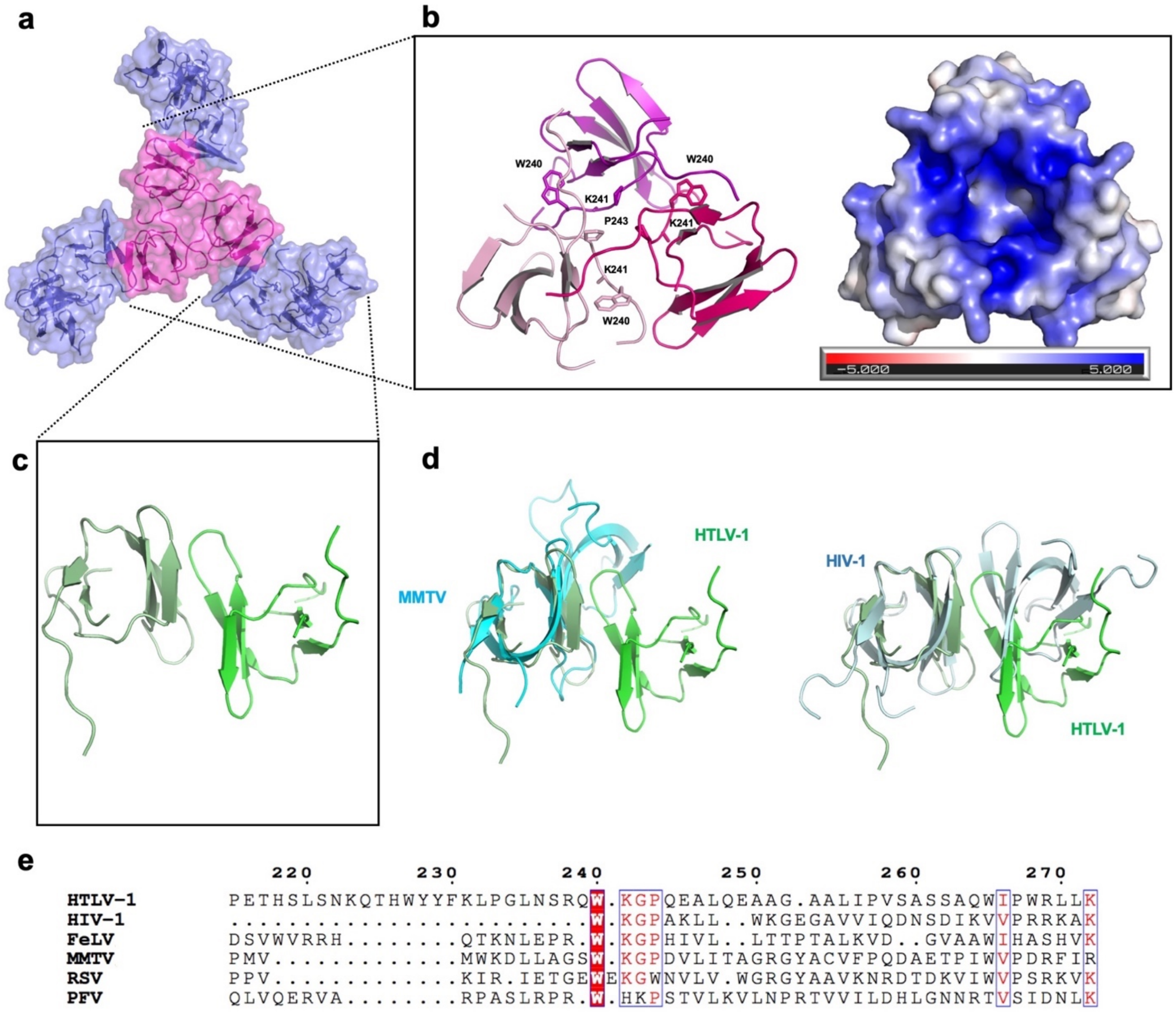

Supplementary Figure 10 | X-ray crystal structure of HTLV-1 IN/CTD. a, A propeller-like trimer of IN/CTD dimers crystal packing of HTLV-1 IN/CTD leads to a canonical dimeric CTD structure (**c**) as well as a novel trimeric species (**b**). **b**, Curiously, the IN/CTD crystal packing creates a stable trimeric interface with a highly positively-charged core, involving residues Trp240-Pro244 – a motif known to be conserved across INs from all retroviral genera (see panel E)[15]. Trimeric forms of IN have not been observed before and although likely not important for the catalytic function of IN, could be relevant to its other functions, such as vRNA binding[16]. The stable trimeric interface harbours strong positive charge in its centre (right, as calculated with the APBS PyMol plugin[17]), neutralised by water molecules. Red signifies negative charge, blue signifies positive charge. **c**, Dimeric packing of HTLV-1 IN/CTD. **d**, HTLV-1 IN/CTD dimers (green) compared to respective dimers observed in crystal and cryo-EM structures of Mouse Mammary Tumour Virus (MMTV) (cyan) and HIV-1 (light blue) INs. **e**, Alignment of IN/CTD sequences from different retroviral genera. The "WKGP motif"

represents the most intra-genus and inter-genus conserved region of IN/CTD. Red background indicates absolute conservation, red font indicates incomplete conservation. Alignment was conducted with CLUSTALW [12] and visualised in ESPRIPT[13]

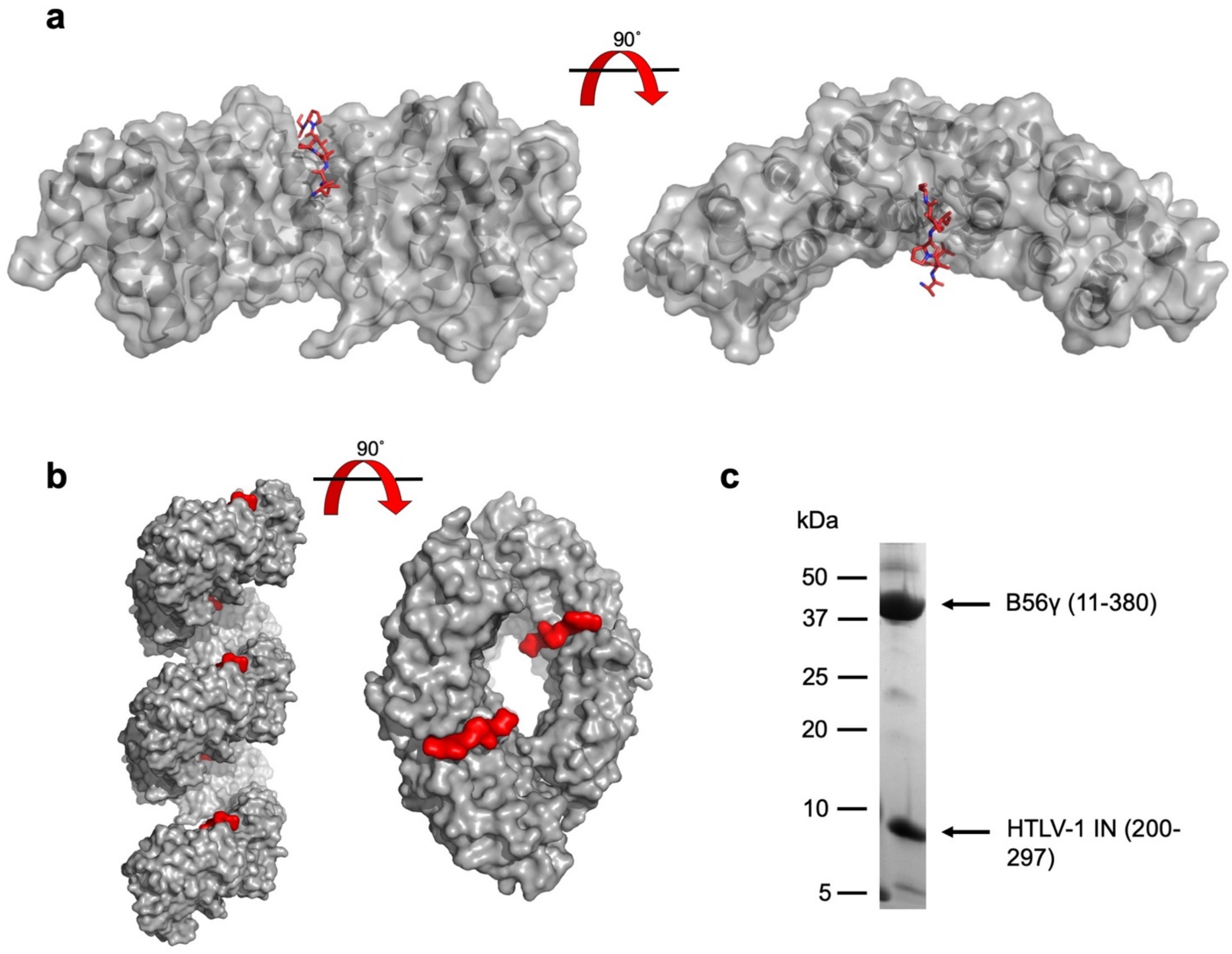

Supplementary Figure 11 | Structure of HTLV-1 IN (200-297) in complex with B56γ. a, Overall crystal structure with B56γ (grey) seen in complex with the CCD-CTD linker region of HTLV-1 IN (red). **b**, Crystal packing creates helical arrangements of B56γ, creating large solvent channels. The resolved, SLiM-containing fragment of IN (red) is positioned facing the lumen of the helix. The remaining, unresolved part of HTLV-1 IN (200-297) is therefore likely to be disordered and contained in the solvent channels created by this crystal packing. **c**, About 50 crystals of the same condition used for diffraction were harvested, crushed, dissolved in running buffer and analysed on SDS-PAGE. This would indicate that despite the majority of HTLV-1 IN (200-297) density missing, the protein in its intact form is present in the crystal. Source data are provided as a Source Data file.

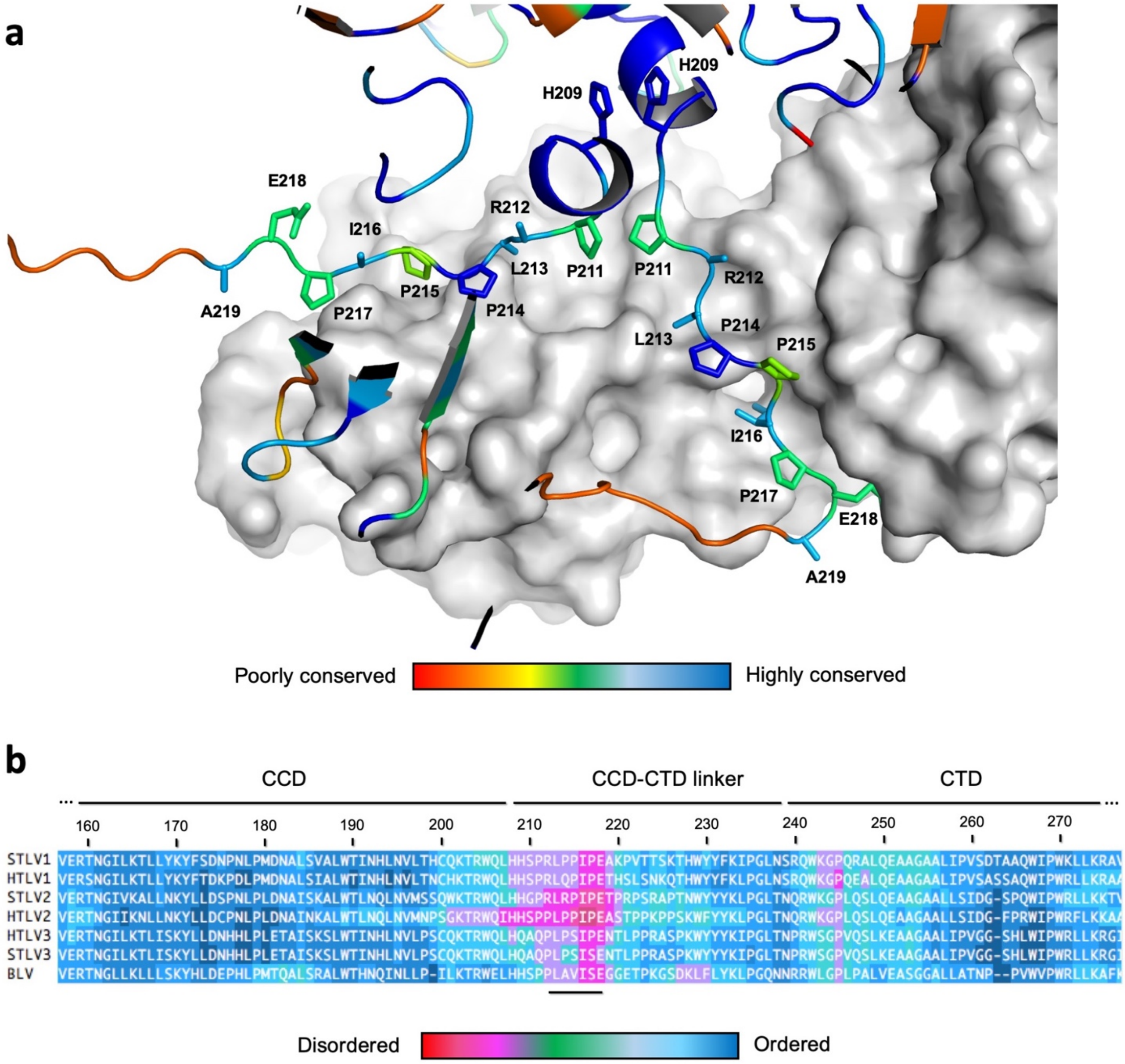

Supplementary Figure 12 | Sequence conservation and intrinsic disorder analysis of STLV-1 IN. a, Sequence conservation between deltaretroviral IN linker regions was projected on the STLV-1 : B56γ intasome structure, showing high conservation in the SLiM region engaged in B56γ interactions in all resolved IN subunits. Residues $_{213}$LPPIPE$_{218}$ comprise the conserved LxxIxE interaction motif. Sequence conservation is coloured from blue (high conservation) to red (no conservation). Figure was prepared with Alebrijes 2.1 (https://github.com/mbarski/Alebrijes) **b**, Disorder prediction on aligned deltaretroviral INs shows that the SLiM-containing IN CCD-CTD linker is intrinsically disordered (LxxIxE motif, underlined). Conservation of disorder in this region among deltaretroviral INs is also clearly

visible. Red indicates high disorder, dark blue - absence of disorder. Analysis was conducted with BASILIScan 1.3[18].

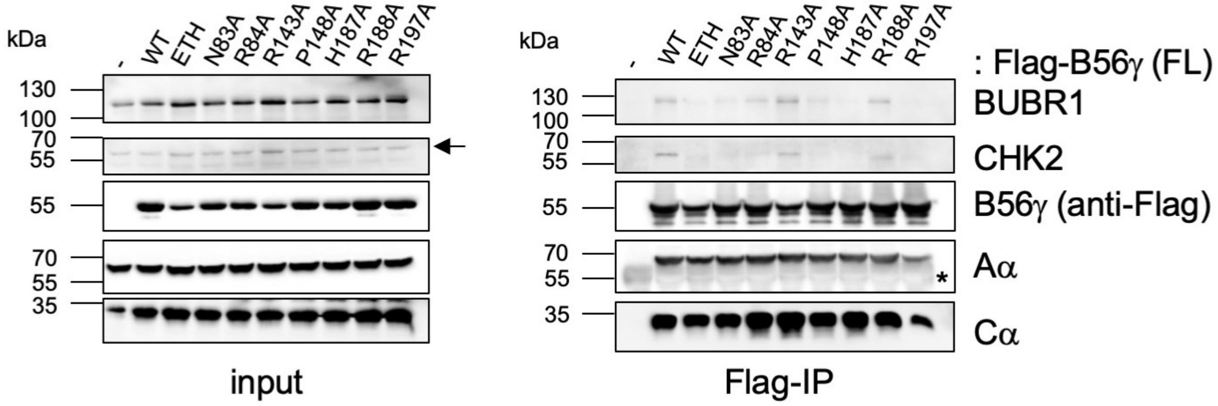

Supplementary Figure 13 | Co-immunoprecipitation (IP) of endogenous PP2A-B56γ substrates. Extracts from 293T cells transiently transfected with Flag-tagged full-length B56γ WT or mutant versions were immunoprecipitated with anti-Flag antibodies. Binding to endogenous substrates (BUBR1 and CHK2) as well as formation of the holo-enzyme was investigated by Western blot. Antibodies used are indicated to the right of the blots, Mw markers are indicated to the left. Mutants are shown on top of the blots. ETH = B56γ(E78A/T81A/H82A). As a negative control empty vector was transfected into 293T cells (-). Input and Flag-IP samples are shown in the left and the right panel, respectively. *indicates cross-reaction of the secondary anti-rat antibody with the mouse anti-Flag antibody used during IP. Arrow points to the CHK2 band. This is a representative of three biological replicates. Source data are provided as a Source Data file.

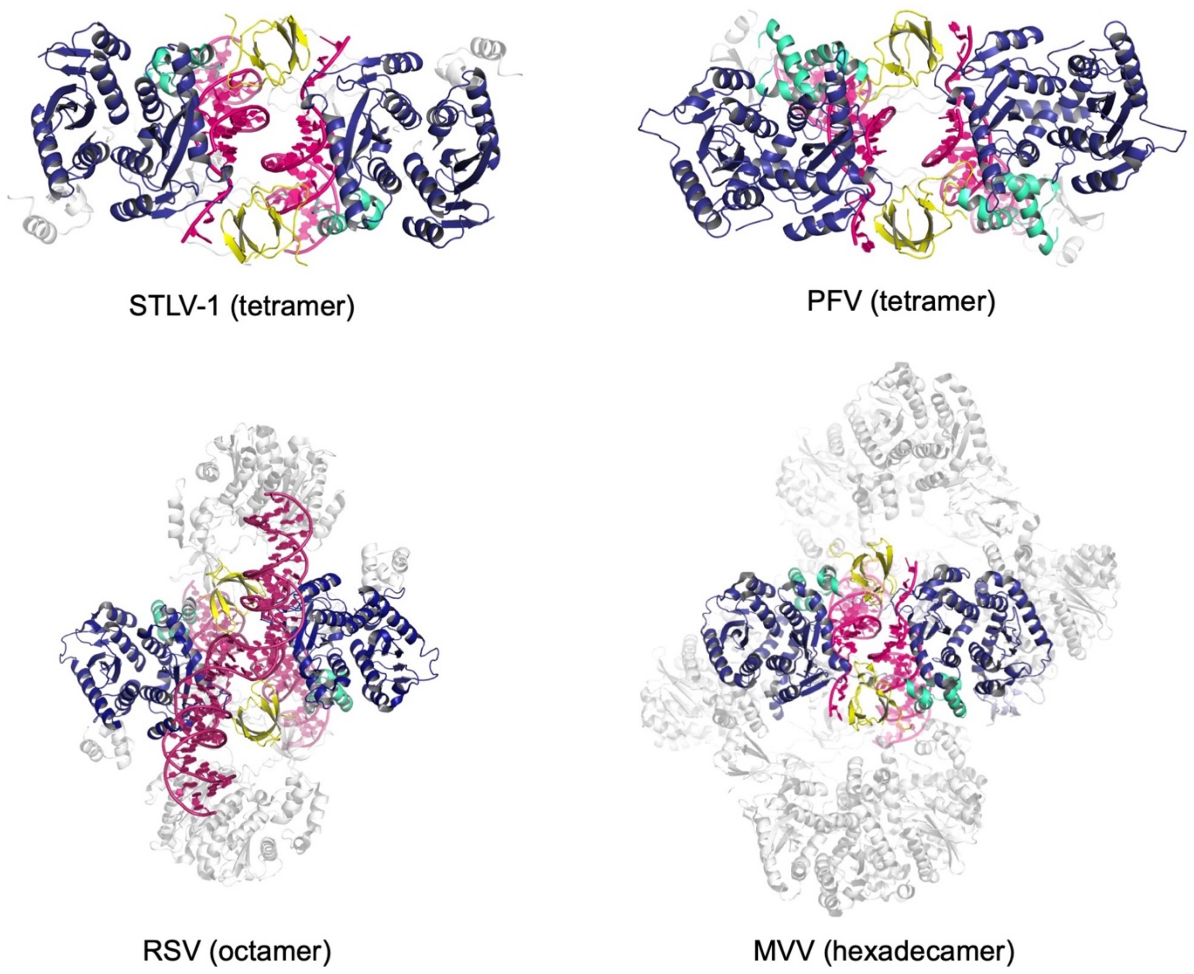

Supplementary Figure 14 | The STLV-1 intasome structure as compared to intasomes from other retroviral genera. Structures of intasome complexes display surprising variability among the different retroviral genera. Although the oligomeric assemblies differ significantly, the conserved intasome core (CIC), coloured non-grey above, is largely unchanged. The structure of STLV-1 intasome is closest to that of PFV. B56γ was removed for clarity. IN/NTDs are shown in cyan, IN/CCDs in blue, IN/CTDs in yellow and vDNA in pink, and all residues outside of the CIC were coloured in semi-transparent grey. PDB accession codes are as follows: 6Z2Y (STLV-1), 3L2Q (Prototype Foamy Virus, PFV), 5EJK (Rous Sarcoma Virus, RSV), 5M0Q (Maedi-Visna Virus, MVV).

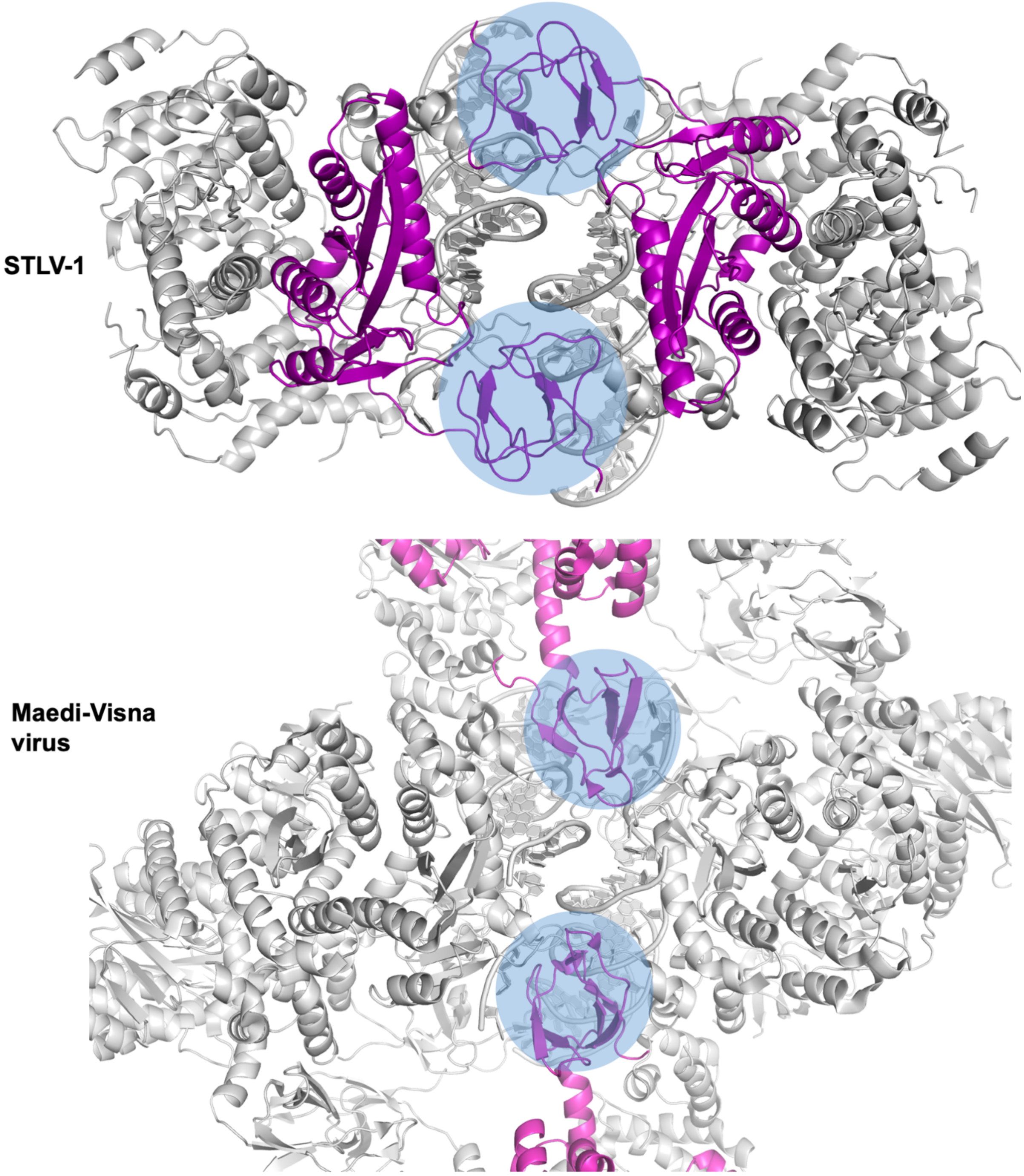

Supplementary Figure 15 | Positioning of the synaptic CTD domains in intasome structures. The extended nature of the CCD-CTD linkers of STLV-1 IN allow for positioning of synaptic CTDs in *cis* (top), while INs with shorter, or coiled CCD-CTD linkers (for example MVV, bottom) require positioning of the synaptic CTD in *trans*, engaging flanking IN subunits and leading to a larger oligomeric state of the intasome. For clarity the B56γ host factor was removed from the STLV-1 intasome structure (top). PDB accession codes: 6Z2Y (STLV-1), and 5M0Q (MVV).

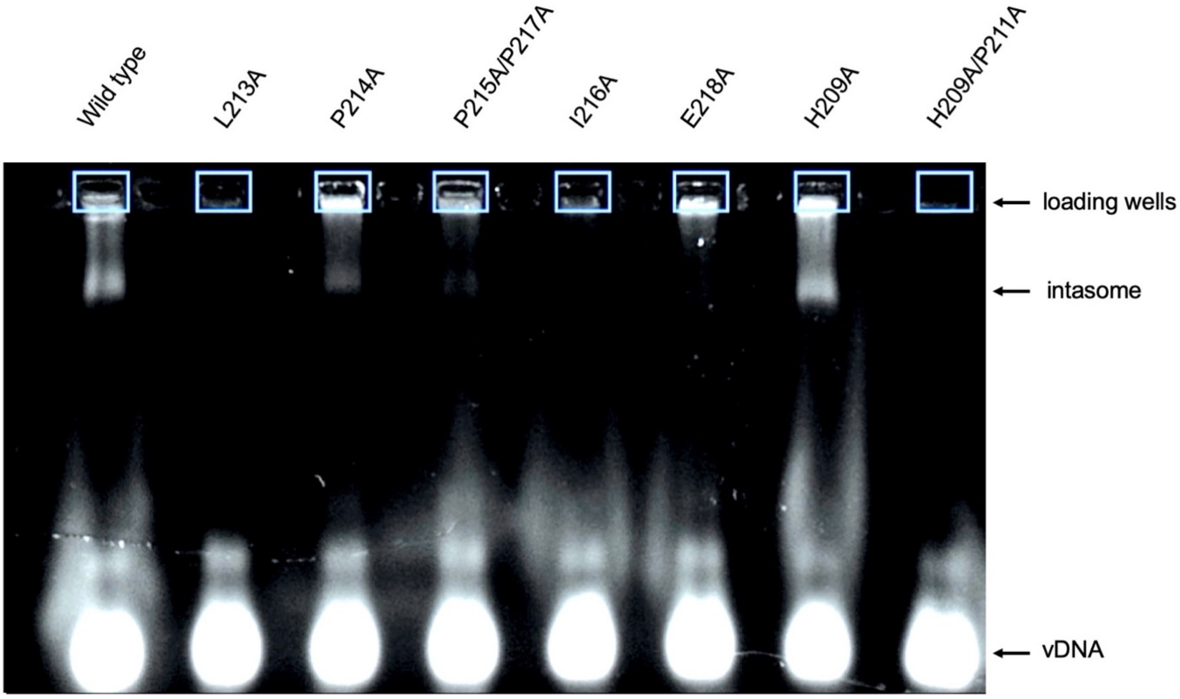

Supplementary Figure 16 | Electrophoretic mobility shift assays analysis of STLV-1 IN mutants. Representative image of EMSA used for quantification shown in Fig. 2. As seen in Supplementary Figure 2, LEDGF/ΔIBD-B56γ is crucial for intasome assembly *in vitro*. Mutations of IN residues seen involved in binding to B56γ in the cryo-EM structure affect assembly of the intasome. Atto680-labelled vDNA (30 bp) was used to visualise the DNA on a 3% low melting point agarose gel. Experiment was conducted in triplicate for quantification shown in Fig. 2.

Supplementary tables

Supplementary Table 1. Data collection and refinement statistics of HTLV-2 IN CCD crystal structures

	IN CCD-Mg^{2+}	IN CCD-Mg^{2}	IN CCD-Ca^{2+}
**Data collection**			
Space group	$P4_332$	C121	$P4_332$
Cell dimensions			
a, *b*, *c* (Å)	115.9, 115.9, 115.9	185.3, 89.2, 65.5	112.1, 112.1, 112.1
α, β, γ (°)	90, 90, 90	90, 103, 90	90, 90, 90
Resolution (Å)	40.98 - 2.29 (2.37 - 2.29)	58.54 – 2.45 (2.55 – 2.45)	50.12 – 2.40 (2.49 – 2.40)
R_{merge} (%)	2.3 (29)	24.2 (38.3)	2.8 (31)
$<I / \sigma(I)>$	24.2 (2.5)	3.0 (1.2)	13.8 (1.8)
Completeness (%)	99.9 (99.9)	100 (100)	100 (100)
Redundancy	1.8 (1.9)	3.9 (3.9)	1.8 (1.9)
**Refinement**			
Resolution (Å)	2.29	2.45	2.40
No. reflections	22,653 (2,228)	149,376 (16,967)	17,832 (1,872)
R_{work} / R_{free}	19.66/23.91	24.99/27.64	24.71/28.19
No. atoms			
Protein	1,195	4,640	1,190
Ligand/ion	1	4	1
Water	59	100	28
B-factors (Å^2)			
Protein	38.95	70.57	55.01
Ligand/ion	53.35	72.75	69.99
Water	64.19	81.82	84.59
R.m.s. deviations			
Bond lengths (Å)	0.017	0.011	0.005
Bond angles (°)	2.047	1.42	0.860

The values in parenthesis refer to the highest resolution shell.

[a] $R_{merge} = \frac{\sum_{hkl}\sum_i |I(hkl;i) - <I(hkl)>|}{\sum_{hkl}\sum_i I(hkl;i)}$, where I(hkl; i) is the intensity of an individual measurement of a reflection and <I(hkl)> is the average intensity of that reflection.

[b] $R_{work} = \frac{\sum_{hkl} F_0 - F_c}{\sum_{hkl} F_0}$, where F_o and F_c are the observed and calculated structure factors, respectively.

[c] R_{free} is R_{work} with 5% of the observed reflections removed before refinement.

Supplementary Table 2. Data collection and refinement statistics of HTLV-1 IN CTD and HTLV-1 IN(200-297) : B56γ crystal structures

	**HTLV-1 IN CTD**	**HTLV-1 IN(200-297)-B56γ**
**Data collection**		
Space group	$I4_132$	$P4_32_12$
Cell dimensions		
a, *b*, *c* (Å)	97.41, 97.41, 97.41	58.77, 58.77, 321.15
α, β, γ (°)	90, 90, 90	90, 90, 90
Resolution (Å)	39.80 – 1.80 (1.85 – 1.80)	80.29 – 3.16 (3.38 – 3.16)
R_{merge} (%)	7.7 (71)	16.7 (48.9)
$<I / (\sigma I)>$	7.4 (1.8)	3.3 (1.5)
Completeness (%)	100 (100)	96.7 (94.4)
Redundancy	5.4 (5.6)	1.4 (1.2)
**Refinement**		
Resolution (Å)	1.8	3.16
No. reflections	41,483 (2,513)	14,094 (2,025)
R_{work} / R_{free}	17.71/24.64	22.7/29.4
No. atoms		
Protein	840	2,674
Ligand/ion	0	0
Water	57	46
B-factors		
Protein	55.00	53.90/79.71
Ligand/ion	N/A	N/A
Water	71.53	40.47
R.m.s. deviations		
Bond lengths (Å)	0.012	0.0216
Bond angles (°)	1.861	1.98

The values in parenthesis refer to the highest resolution shell.

[a] $R_{merge} = \frac{\sum_{hkl}\sum_i |I(hkl;i) - <I(hkl)>|}{\sum_{hkl}\sum_i I(hkl;i)}$, where I(hkl; i) is the intensity of an individual measurement of a reflection and <I(hkl)> is the average intensity of that reflection.

[b] $R_{work} = \frac{\sum_{hkl} F_0 - F_c}{\sum_{hkl} F_0}$, where F_o and F_c are the observed and calculated structure factors, respectively.

[c] R_{free} is R_{work} with 5% of the observed reflections removed before refinement.

Supplementary Table 3. Comparison of CCD-CTD linker lengths and respective oligomeric state of selected intasome structures and free IN of retroviral genera ordered according to linker length and its topology

Genus	Virus	CCD-CTD linker length	Intasome oligomer	Uncomplexed IN	CCD-CTD linker topology	Reference
Spumavirus	PFV	50	Tetramer	Monomer	Extended coil	[19]
Deltaretrovirus	STLV-1	19	Tetramer	Dimer	Extended coil	This study
Lentivirus	MVV	20	Hexadecamer	Octamer	Helix	[20]
Betaretrovirus	MMTV	8	Octamer	Dimer	Short coil	[21]
Alpharetrovirus	RSV	8	Octamer	Dimer	Short coil	[22]

Supplementary Table 4. Primers used for STLV-1 IN strand-transfer assays

Name	Sequence
Mar_U5_S30UP	TCTCTCCGGGAGAGAAGCGCCAAACACA
Mar_U5_S30B	ACTGTGTTTGGCGCTTCTCTCCCGGAGAGA

Supplementary Table 5. Cryo-EM reconstruction and STLV-1 intasome refinement statistics

	STLV-1 intasome	
**Data collection**	OH subset	GO subset
Voltage (keV)	300	300
Cumulative exposure ($e^{-}/Å^{2}$)	34	34
Number of frames per movie	30	30
Defocus range (µm)	-1.6 to -3.6	-1.6 to -3.6
Pixel size (Å)	1.09	1.09
**Particle classification**		
Initial particle images (no.)	2,198,454	2,157,654
Particles after 2D classification	599,700	493,665
Particles after 3D classification	94,517	67,404
**Merged 3D reconstruction**		
Particles used	161,914	
Symmetry imposed	C2	
Software for reconstruction	Relion-3.1	
Overall resolution (Å)[a]	3.37	
Software for density modification and map sharpening	Resolve(Phenix-1.18-3845) and Relion-3.1	
EMDB accession code	EMD-11052	
**Model Refinement**		
Final model (PDB accession code)	6Z2Y	
Model composition		
Non-hydrogen atoms	14,998	
Protein residues	1,664	
DNA	80	
Zn^{2+}	4	
B factors ($Å^{2}$)		
Protein	31.31	
DNA	49.12	
Zn^{2+}	38.96	
**Model validation**		
MolProbity score	1.42	
Clashscore	3.63	
EMringer score	2.88	
Poor rotamers (%)	0.69	
R.m.s. deviations		
Bond lengths (Å)	0.004	
Bond angles (°)	0.571	
Ramachandran plot:		
Favored (%)	96.10	
Allowed (%)	100	
Disallowed (%)	0	

[a]Based on the FSC of 0.143 between half-sets

Supplementary Table 6. Primers used to clone constructs for recombinant protein expression

Primer name	Primer sequence
GM110	CCGGGTCGACTCAGCCGTGGTGCTGGTGGTC
GM130	GGCCCAATTGCGCCGGGGCCTCTTGCC
GM142	GCTTGAATTCATGGTGGTGGATGCGGC
GNM283	GGCCGTCGACTCAGGTCTTTGAGTTGCGG
GNM367	GGCCGTCGACCTAGCGGCCGTCCTGGG
GNM378	GGCCGAATTCTGCCACAAGACCCGGTGGC
GNM380	GGCCACCGGTATGTTGACATGTAATAAAGCGGGC
GNM641	CCGGGGATCCATGACTCGCGATTTCAAACC
GNM747	GGCCGAATTCCAACTTTCTCCTGCCAAACTACATAGTTTTACTC
GNM748	GGCCCTCGAGTTACCCATGGTGTTGGTGGTCTCTTTC
GNM750	CTGGGTAAATAGGCTCTGTGATCACATTCCGATTAGCGGCGATATATGCTACCATTTCAC
GNM751	CAGCTTGTTTATGAATTTTTCTTAGCATTTTTAGAGTCTCCAGATTTCCAACCTAATATAGCG
GNM752	CGCTATATTAGGTTGGAAATCTGGAGACTCTAAAAATGCTAAGAAAAATTCATAAACAAGCTG
GNM769	GGCCGAATTCCAGCTGAGTCCGGCAAAACTGCATAG
GNM769	GGCCGAATTCCAGCTGAGTCCGGCAAAACTGCATAG
GNM770	GGCCCTCGAGTTAACCATGATGCTGATGATCACGTTC
GNM771	GCTGCATCATAGTCCGCGTGCGCCTCCGATTCCGGAAGCAAAACC
GNM772	GGTTTTGCTTCCGGAATCGGAGGCGCACGCGGACTATGATGCAGC
GNM773	GCATCATAGTCCGCGTCTGGCTCCGATTCCGGAAGCAAAACC
GNM774	GGTTTTGCTTCCGGAATCGGAGCCAGACGCGGACTATGATGC
GNM775	CATCATAGTCCGCGTCTGCCTGCGATTGCGGAAGCAAAACCGGTTACCACC
GNM776	GGTGGTAACCGGTTTTGCTTCCGCAATCGCAGGCAGACGCGGACTATGATG
GNM777	CATAGTCCGCGTCTGCCTCCGGCTCCGGAAGCAAAACCGGTTACC
GNM778	GGTAACCGGTTTTGCTTCCGGAGCCGGAGGCAGACGCGGACTATG
GNM779	GTCCGCGTCTGCCTCCGATTCCGGCAGCAAAACCGGTTACCACCAG
GNM780	CTGGTGGTAACCGGTTTTGCTGCCGGAATCGGAGGCAGACGCGGAC
GNM824	GAATCGGAGGCAGACGCGGACTAGCATGCAGCTGCCAACGGGTTTTC
GNM826	CCGGAATCGGAGGCAGACGCGCACTAGCATGCAGCTGCCAACGGGTTTTC
GNM829	GCTGCATGCTAGTCCGCGTCTGCCTCCGATTC
GNM830	GCTGCATGCTAGTGCGCGTCTGCCTCCGATTCCGG
GNM833	ATGGTAGAATATATCACCCATAATGCGAATGTGATCACAGAGCCTATTTACCCAGAAG
GNM834	CTTCTGGGTAAATAGGCTCTGTGATCACATTCGCATTATGGGTGATATATTCTACCAT
GNM837	GTTTATGAATTTTTCTTAAGATTTTTAGAGTCTGCAGATTTCCAACCTAATATAGCG

GNM838	CGCTATATTAGGTTGGAAATCTGCAGACTCTAAAAATCTTAAGAAAAATTCATAAAC
GNM844	GGTAGAATATATCACCCATGCTCGGAATGTGATCACAGAGC
GNM845	GCTCTGTGATCACATTCCGAGCATGGGTGATATATTCTACC
JM1	GATCCCATCACCATCACCACCATGGCAGCGGCCTGGAAGTGCTGTTTCAAGGCCCGG
JM2	AATTCCGGGCCTTGAAACAGCACTTCCAGGCCGCTGCCATGGTGGTGATGGTGATGG
JM3	GGCCCTCGAGCTAGCGGCCGTCCTGGG
MB054	TATGTCGACTCAAGCAGGTCCGCCC
MB094	TATGTCGACTCAGGTAGAGGCTTCAG
MB114	ATAGAATTCCACTGGTACTACTTCAAG

Supplementary References

1 Maertens, G. N. B'-protein phosphatase 2A is a functional binding partner of delta-retroviral integrase. *Nucleic Acids Res* **44**, 364-376 (2016).

2 Maertens, G. N., El Messaoudi-Aubert, S., Elderkin, S., Hiom, K. & Peters, G. Ubiquitin-specific proteases 7 and 11 modulate Polycomb regulation of the INK4a tumour suppressor. *EMBO J* **29**, 2553-2565 (2010).

3 Winter, G., Lobley, C. M. & Prince, S. M. Decision making in xia2. *Acta Crystallogr D Biol Crystallogr* **69**, 1260-1273 (2013).

4 Kabsch, W. Xds. *Acta Crystallogr D Biol Crystallogr* **66**, 125-132 (2010).

5 Potterton, L. *et al.* CCP4i2: the new graphical user interface to the CCP4 program suite. *Acta Crystallogr D Struct Biol* **74**, 68-84 (2018).

6 Evans, P. R. & Murshudov, G. N. How good are my data and what is the resolution? *Acta Crystallogr D Biol Crystallogr* **69**, 1204-1214 (2013).

7 McCoy, A. J. Solving structures of protein complexes by molecular replacement with Phaser. *Acta Crystallogr D Biol Crystallogr* **63**, 32-41 (2007).

8 Adams, P. D. *et al.* PHENIX: a comprehensive Python-based system for macromolecular structure solution. *Acta Crystallogr D Biol Crystallogr* **66**, 213-221 (2010).

9 Terwilliger, T. C. *et al.* Iterative model building, structure refinement and density modification with the PHENIX AutoBuild wizard. *Acta Crystallogr D Biol Crystallogr* **64**, 61-69 (2008).

10 Magnusdottir, A. *et al.* The structure of the PP2A regulatory subunit B56 gamma: the remaining piece of the PP2A jigsaw puzzle. *Proteins* **74**, 212-221 (2009).

11 Graham, F. L. & van der Eb, A. J. A new technique for the assay of infectivity of human adenovirus 5 DNA. *Virology* **52**, 456-467 (1973).

12 Notredame, C. Computing multiple sequence/structure alignments with the T-coffee package. *Curr Protoc Bioinformatics* **Chapter 3**, Unit 3 8 1-25 (2010).

13 Robert, X. & Gouet, P. Deciphering key features in protein structures with the new ENDscript server. *Nucleic Acids Res* **42**, W320-324 (2014).

14 Tan, Y. Z. *et al.* Addressing preferred specimen orientation in single-particle cryo-EM through tilting. *Nat Methods* **14**, 793-796 (2017).

15 Lutzke, R. A., Vink, C. & Plasterk, R. H. Characterization of the minimal DNA-binding domain of the HIV integrase protein. *Nucleic Acids Res* **22**, 4125-4131 (1994).

16 Kessl, J. J. *et al.* HIV-1 Integrase Binds the Viral RNA Genome and Is Essential during Virion Morphogenesis. *Cell* **166**, 1257-1268 (2016).

17 Jurrus, E. *et al.* Improvements to the APBS biomolecular solvation software suite. *Protein Sci* **27**, 112-128 (2018).

18 Barski, M. BASILIScan: a tool for high-throughput analysis of intrinsic disorder patterns in homologous proteins. *BMC Genomics* **19**, 902, doi:10.1186/s12864-018-5322-5 (2018).

19 Hare, S., Gupta, S. S., Valkov, E., Engelman, A. & Cherepanov, P. Retroviral intasome assembly and inhibition of DNA strand transfer. *Nature* **464**, 232-236 (2010).

20 Ballandras-Colas, A. *et al.* A supramolecular assembly mediates lentiviral DNA integration. *Science* **355**, 93-95 (2017).

21 Ballandras-Colas, A. *et al.* Cryo-EM reveals a novel octameric integrase structure for betaretroviral intasome function. *Nature* **530**, 358-361 (2016).

22 Yin, Z. *et al.* Crystal structure of the Rous sarcoma virus intasome. *Nature* **530**, 362-366 (2016).
