# Supplementary material for: Cryo-EM structure of the deltaretroviral intasome in complex with the PP2A regulatory subunit B56γ
Source: Nat Commun. 2020 Oct 7;11:5043. doi: 10.1038/s41467-020-18874-y (PMC7542444; doi:10.1038/s41467-020-18874-y)
Supplement: Supplementary file 3 — Reporting Summary [file 41467_2020_18874_MOESM3_ESM.pdf]

nature research

Corresponding author(s): Maertens, Goedele N.

Last updated by author(s): 27/08/20

Reporting Summary

Nature Research wishes to improve the reproducibility of the work that we publish. This form provides structure for consistency and transparency in reporting. For further information on Nature Research policies, see our Editorial Policies and the Editorial Policy Checklist.

Statistics

For all statistical analyses, confirm that the following items are present in the figure legend, table legend, main text, or Methods section.

n/a	Confirmed	
☐	☒	The exact sample size (*n*) for each experimental group/condition, given as a discrete number and unit of measurement
☐	☒	A statement on whether measurements were taken from distinct samples or whether the same sample was measured repeatedly
☐	☒	The statistical test(s) used AND whether they are one- or two-sided<br>*Only common tests should be described solely by name; describe more complex techniques in the Methods section.*
☒	☐	A description of all covariates tested
☒	☐	A description of any assumptions or corrections, such as tests of normality and adjustment for multiple comparisons
☐	☒	A full description of the statistical parameters including central tendency (e.g. means) or other basic estimates (e.g. regression coefficient) AND variation (e.g. standard deviation) or associated estimates of uncertainty (e.g. confidence intervals)
☐	☒	For null hypothesis testing, the test statistic (e.g. *F*, *t*, *r*) with confidence intervals, effect sizes, degrees of freedom and *P* value noted<br>*Give P values as exact values whenever suitable.*
☒	☐	For Bayesian analysis, information on the choice of priors and Markov chain Monte Carlo settings
☒	☐	For hierarchical and complex designs, identification of the appropriate level for tests and full reporting of outcomes
☒	☐	Estimates of effect sizes (e.g. Cohen's *d*, Pearson's *r*), indicating how they were calculated

Our web collection on statistics for biologists contains articles on many of the points above.

Software and code

Policy information about availability of computer code

Data collection	Cryo-EM movies were recorded using EPU 1.9.0 software (Thermo Fisher Scientific). A high-quality homology model of the STLV-1 IN/NTD was generated by SWISS-MODEL server.<br>Gel images of EMSAs were collected using the Azure c600 imager, Coomassie stained and ethidium bromide stained gels were acquired using the BioRad GelDoc XR+.
Data analysis	Micrograph movie frames were aligned and summed with dose weighting applied as implemented in MotionCor2, and the contrast transfer function (CTF) parameters were estimated from the frame sums using Gctf-v1.06. A small subset of micrographs were picked manually with EMAN2 boxer and subjected to reference-free classification in Relion 2 to generate initial 2D class averages. These were used as templates for picking the entire datasets with Gautomatch v0.56 (http://www.mrc-lmb.cam.ac.uk/kzhang/). The particles extracted in Relion-3.0, binned by a factor of 2, were subjected to two rounds of reference free 2D classification in CryoSPARC 2. 3D classification into was done in Relion-3.0 without imposing symmetry, with an initial model generated in CryoSPARC-2.3D reconstruction, followed by Bayesian polishing, per-particle defocus and beam tilt refinement, as implemented in Relion-3.1, resulted in the final map with minimal anisotropy. 3D-FSC sphericity index of the final map was 0.967. Gold-standard Fourier shell correlation (FSC) = 0.143 criterion was used to estimate resolutions of the 3D reconstructions. Local resolution of the cryoEM map was estimated using Blocres from the Bsoft software package. The quality of the cryoEM map was marginally improved using Resolve density modification procedure implemented in Phenix 1.18-3845. X-ray crystal structures were docked into resulting cryoEM maps as rigid bodies in Chimera. Ab initio building residues not present in docked models but resolved in the cryoEM density and manual refitting of docked models was conducted in Coot 0.8.9.2. The initial model was subjected to molecular dynamics structural fitting using Namdinator. Real space refinement using Phenix version 1.18-3845. Quality of the final atomistic model was assessed with MolProbity and EMRinger.<br>For the crystal structures: Data were indexed and integrated in Xia2 using XDS. Datasets were scaled and merged in CCP4i2 Aimless, with the resolution limit adjusted accordingly until satisfactory signal-to-noise and completeness were reached. Unit cell composition was estimated from Matthew's coefficient. Phases were obtained by molecular replacement in PHASER through the PHENIX 1.18-3845 software suite. PHENIX Autobuild was successful in building the majority of residues in all chains. The remaining residues were added manually in Coot 0.8.9.2 and the model was refined in Refmac 5.8. SEC-MALLS data was analysed using ASTRA 7.1.0.29 software.

Quantification of bands in EMSA gels was done using ImageJ software and of the Coomassie and ethidium bromide stained gels the built in BioRad software was used to determine the density of the bands using the raw image. Graphpad Prism 8 was used to calculate means, standard deviations and assess statistical significance (student t-test).

For manuscripts utilizing custom algorithms or software that are central to the research but not yet described in published literature, software must be made available to editors and reviewers. We strongly encourage code deposition in a community repository (e.g. GitHub). See the Nature Research guidelines for submitting code & software for further information.

Data

Policy information about availability of data

All manuscripts must include a data availability statement. This statement should provide the following information, where applicable:

- Accession codes, unique identifiers, or web links for publicly available datasets
- A list of figures that have associated raw data
- A description of any restrictions on data availability

The crystal structures have been deposited with the Protein Data Bank and are available under the following identifiers: HTLV-2/CCD-Mg2+ (dimeric form): 6QBV; HTLV-2/CCD-Mg2+ (trimeric form): 6QBT; HTLV-2/CCD-Ca2+ (dimeric form): 6QBW; HTLV-1/CTD: 6TJU; HTLV-1 IN(200-297)-B56gamma: 6TOQ. The cryoEM structure has been deposited with the Protein Data Bank and EMDB and are available under the following identifiers 6Z2Y and EMD-11052. The authors declare that all other data supporting the findings of this study are available within the paper, its supplementary information files, and the Source data (for Supplementary Figure 2 and 13, and the raw data used to make bar graphs in Figure 2) provided with this paper. Correspondence and requests for materials should be addressed to G.N.M. (g.maertens@imperial.ac.uk).

Field-specific reporting

Please select the one below that is the best fit for your research. If you are not sure, read the appropriate sections before making your selection.

☒ Life sciences ☐ Behavioural & social sciences ☐ Ecological, evolutionary & environmental sciences

For a reference copy of the document with all sections, see nature.com/documents/nr-reporting-summary-flat.pdf

Life sciences study design

All studies must disclose on these points even when the disclosure is negative.

Sample size	Sample sizes of the cryo-EM data sets are included in Supplementary Table 5 and in the processing flow chart in Supplementary Figure 5
Data exclusions	Cryo-EM micrographs were excluded based on drift and resolution criteria. Particles were excluded from final maps by 2D and 3D classification. Details are included in Supplementary Table 5 and Supplementary Figure 5.
Replication	Experiments were repeated at least three times. Exact numbers are given in the "Reproducibility and Statistics" section in Methods
Randomization	N/A not a clinical study
Blinding	N/A not a clinical study

Reporting for specific materials, systems and methods

We require information from authors about some types of materials, experimental systems and methods used in many studies. Here, indicate whether each material, system or method listed is relevant to your study. If you are not sure if a list item applies to your research, read the appropriate section before selecting a response.

Materials & experimental systems

n/a	Involved in the study	
☐	☒	Antibodies
☐	☒	Eukaryotic cell lines
☒	☐	Palaeontology and archaeology
☒	☐	Animals and other organisms
☒	☐	Human research participants
☒	☐	Clinical data
☒	☐	Dual use research of concern

Methods

n/a	Involved in the study	
☒	☐	ChIP-seq
☒	☐	Flow cytometry
☒	☐	MRI-based neuroimaging

Antibodies

Antibodies used	horse radish peroxidase (HRP) conjugated mouse anti-Flag antibody (1: 2,000, clone M2, Sigma, A8592, lot number: SLBB9238),

Antibodies used	rabbit anti-BUBR1 (1:1,000, Bethyl Laboratories, A300-365A-T, lot number A300-386A-T-2),<br>rabbit anti-CHK2 (1:200, Santa Cruz Biotech, H-300, sc-9064, lot number L2405),<br>rat anti-Aalpha (1:2,000 clone 6G3, Insight Biotechnology, sc-56954, lot number F0413),<br>mouse anti-Calpha (1:2,000, clone 46, Becton-Dickinson, 610556, lot number 58002)<br>HRP conjugated donkey anti-rabbit (GE Life Sciences, GE NA934, lot number LNA934V/AG),<br>rabbit anti-rat (Abcam, ab6734, lot number GR36157-10) and<br>rat anti-mouse (Abcam, ab131368, lot number GR3235649-7)
Validation	Validation by provider:<br>BUBR1 antibody: validation by IP/western blot performed using ReliaBLOT reagents<br>anti-Calpha: by western blot on A431 cell lysate<br>HRP conjugated anti-Flag: detects a single band of protein on a Western blot from an E. coli crude cell lysate<br>anti-Aalpha validated by western blot analysis of PP2A-Aalpha expression in a range of eukaryotic cell lysates<br>anti-CHK2: validated by western blot analysis of CHK2 expression in HL-60 and HeLa cells

Eukaryotic cell lines

Policy information about cell lines

Cell line source(s)	HEK293T from ATCC
Authentication	not authenticated
Mycoplasma contamination	Cells were negative for mycoplasma contamination
Commonly misidentified lines<br>(See ICLAC register)	no commonly misidentified cell lines were used
